# Cocaine-Induced Changes in Low-Dimensional Attractors of Local Field Potentials in Optogenetic Mice

**DOI:** 10.3389/fncom.2018.00002

**Published:** 2018-01-31

**Authors:** Sorinel A. Oprisan, Julia Imperatore, Jessica Helms, Tamas Tompa, Antonieta Lavin

**Affiliations:** ^1^Department of Physics and Astronomy, College of Charleston, Charleston, SC, United States; ^2^Department of Neuroscience, Medical University of South Carolina, Charleston, SC, United States; ^3^Department of Preventive Medicine, Faculty of Healthcare, University of Miskolc, Miskolc, Hungary

**Keywords:** optogenetics, medial prefrontal cortex, electrophysiology, delay-embedding, non-linear dynamics

## Abstract

Optogenetically evoked local field potential (LFP) recorded from the medial prefrontal cortex (mPFC) of mice during basal conditions and following a systemic cocaine administration were analyzed. Blue light stimuli were delivered to mPFC through a fiber optic every 2 s and each trial was repeated 100 times. As in the previous study, we used a surrogate data method to check that nonlinearity was present in the experimental LFPs and only used the last 1.5 s of steady activity to measure the LFPs phase resetting induced by the brief 10 ms light stimulus. We found that the steady dynamics of the mPFC in response to light stimuli could be reconstructed in a three-dimensional phase space with topologically similar “8”-shaped attractors across different animals. Therefore, cocaine did not change the complexity of the recorded nonlinear data compared to the control case. The phase space of the reconstructed attractor is determined by the LFP time series and its temporally shifted versions by a multiple of some lag time. We also compared the change in the attractor shape between cocaine-injected and control using (1) dendrogram clustering and (2) Frechet distance. We found about 20% overlap between control and cocaine trials when classified using dendrogram method, which suggest that it may be possible to describe mathematically both data sets with the same model and slightly different model parameters. We also found that the lag times are about three times shorter for cocaine trials compared to control. As a result, although the phase space trajectories for control and cocaine may look similar, their dynamics is significantly different.

## 1. Introduction

Pyramidal cells together with spiny stellates constitute more than 70% of the excitatory neural population of the cortex (Feldman, 1984; Bannister, 2005). Pyramidal cells form wide interconnected network spanning layers 2–6 (Bannister, 2005). They serve, among other functions, maintaining prefrontal cortex activity during working memory (Sanchez-Vives and McCormick, 2000) and generate the UP states of persistent network activity (Luczak et al., 2007; Compte et al., 2008).

The pyramidal neurons receive inhibitory inputs from GABA interneurons, of which the most prominent group expresses the calcium-binding protein parvalbumin (PV) (Takahata and Kato, 2008; Casanova and Trippe, 2009; Kana et al., 2011). It is thought that the PV-positive (PV+) neurons projections coordinate the output of local minicolumns (Galarreta and Hestrin, 2001; Sultan et al., 2013). GABAergic interneurons present significant density and morphological heterogeneities across different cortical areas (Rodriguez et al., 2002) in order to regulate the firing rate of pyramidal cells and facilitate information processing (Guidotti et al., 2005; Fuchs et al., 2007; Schmidt and Mirnics, 2015). PV+ interneurons also present a significant heterogeneity of projections to pyramidal cells processes that allow them a fine-tuned functional control of pyramidal cells (DeFelipe and Farinas, 1992). It has been established that projections of PV+ neurons to pyramidal cells soma and proximal dendrites are very effective in modulating their firing rate (Halasy et al., 1996; Booker et al., 2013), whereas synapses on the axons of pyramidal cells can block action potentials (Melchitzky and Lewis, 2003; Henry et al., 2004; Micheva et al., 2016).

It is also believed that PV+ neurons are instrumental in maintaining and/or modulating both beta (15–30 Hz) and gamma (25–40 Hz) rhythms of the brain. It has been shown that beta rhythm activity could be linked to autism, as it coordinates activity across fronto-parietal networks (Schnitzler and Gross, 2005; Peter and Wolf, 2010), and schizophrenia (Liddle et al., 2016). Beta rhythm has also been associated with sensory gating (Michael and Zoe, 2006; Hong et al., 2008; Cheng et al., 2016). GABAergic neurons in beta band are thought to guide early stage development of radial columnar circuits of pyramidal and radial interneurons (Rippon et al., 2007; Casanova and Trippe, 2009). In this respect, GABAergic neurons could also be involved in schizophrenia (Schmidt and Mirnics, 2015), although recent studies (O'Connell et al., 1997; Muraki and Tanigaki, 2015) suggested that this disorder results from embryonic developmental abnormalities rather than from neuronal degenerations.

Gamma band activity has been linked to sensory processing of stimulus characteristics (Kaiser and Lutzenberger, 2003) and is determined by the details of the local circuits involving PV+ fast-spiking interneurons (Cardin et al., 2009; Sohal et al., 2009, 2016). PV+ interneurons are thought to also promote gamma band signal transmission both within (Veit et al., 2017) and between neocortical areas (Cantero and Atienza, 2005), while corresponding abnormalities in this process may contribute to schizophrenia (Lewis et al., 2005; Lewis and Hashimoto, 2007) and autism (Levy, 2007; Orekhova et al., 2007). Since PV+ neurons provide inhibitory modulation, it has been found that a decrease in local inhibition could lead to sensory hypersensitivity and neural hyper-excitability (Gibson et al., 2008; Rotschafer and Razak, 2014; Contractor et al., 2017; Ethridge et al., 2017).

Optogenetic techniques have recently been used for investigating basic questions regarding neural plasticity mechanisms (Iurilli et al., 2012; Eleftheriou et al., 2017; Kim et al., 2017), information processing (Sohal et al., 2009, 2016; Wilson et al., 2012), hippocampal memory formation (Liu et al., 2012; Ramirez et al., 2014), and for the design of neural interface (Rivnay et al., 2017). Optogenetics was also used for investigating complex patterns of behavior, such as feeding (Aponte et al., 2011; Atasoy et al., 2012; Jennings et al., 2013; Chen and Knight, 2016), fear conditioning (Haubensak et al., 2010; Do Monte et al., 2016), and aggression (Lin et al., 2011). Optogenetics gave promising results in restoration of visual functions in blind animals (Lagali et al., 2008; Busskamp et al., 2010; Gelder, 2015), treatment of neural disorders, such as anxiety and depression (Tye et al., 2011, 2013; Allsop et al., 2014), and ameliorating neurodegenerative conditions, such as Parkinson's disease (Gradinaru et al., 2009; Kravitz et al., 2010), and epilepsy (Kokaia et al., 2013; Paz et al., 2013; Peng et al., 2013; Wykes et al., 2016).

We used optogenetic tools to investigate the response of the local network in the medial prefrontal cortex (mPFC) of mice under brief light stimuli. This study used the same knock-in mouse model together with optogenetics and *in vivo* electrophysiology described in detail in Dilgen et al. (2013). The goal was to investigate the effects of acute cocaine on mPFC gamma oscillation and their relationship to more permanent cortical changes of long-term use of stimulants. We previously carried out a similar data mining study on *the same animals* under control conditions (Oprisan et al., 2015). The present study is a continuation of Oprisan et al. (2015) in which the same mice were systemically injected with cocaine. We performed a nonlinear time series analysis of LFPs recorded from PV+ neurons using time reversal asymmetry and false nearest neighbor (FNN) statistics between the original signal and surrogate data (Oprisan et al., 2015) to identify the nonlinearity in the data set.

The local field potential (LFP) is the sum of excitatory and inhibitory dendritic potentials in a small region (approximately 200–400 μm Katzner et al., 2009) around the tip of the electrode (Scherberger et al., 2005; Kajikawa and Schroeder, 2011). As opposed to spike recording through intra/extra-cellular microelectrodes, which represent neural outputs, the LFPs represent inputs and local processing from synaptic activity (Mitzdorf, 1987). Additionally, LFPs are easier to record, which could be useful for practical implementations of control mechanisms similar to deep brain stimulation or brain-computer interfaces (Hatsopoulos and Donoghue, 2009). It has been shown that cognitive processes could modulate the temporal structure of the LFPs (Pesaran et al., 2002; Mehring et al., 2003). Such a temporal structure could be captured by the lag time distribution of delay-embedding method used here.

Oscillatory activity of individual neurons contributes to the observed beta and gamma rhythms of the brain (Buzsáki and Draguhn, 2004; Fujiwara-Tsukamoto and Isomura, 2008; Liddle et al., 2016), they allow task coordination (Kahana et al., 1999), support memory formation and retrieval (Roux and Uhlhaas, 2014), or signal neuropathological conditions (Orekhova et al., 2007; Peter and Wolf, 2010). Ongoing oscillatory activity could be reset by sensory inputs, such as visual (Kambe et al., 2015; Woelders et al., 2017) or auditory (Mercier et al., 2013) stimuli, or by extrinsic stimuli, such as deep brain stimulation (Tass, 2003), or temperature (Rensing and Ruoff, 2002). We measured the phase resetting induced by brief light stimuli using both the autocorrelation (Oprisan, 2013; Oprisan et al., 2015) and the Hilbert's transform (Oprisan, 2017) methods. The phase resetting correction allowed an accurate estimate of the delay (lag) time and embedding dimension of LFP data (Oprisan and Canavier, 2002; Oprisan et al., 2003, 2015).

## 2. Materials and methods

### 2.1. Human search and animal research

A detailed description of the procedures can be found in the first paper of this series (Oprisan et al., 2015) and we only briefly summarize them here. All procedures were done in accordance to the National Institute of Health guidelines as approved by the Medical University of South Carolina Institutional Animal Care and Use Committee.

### 2.2. Experimental protocol

The experimental protocol is the same as in Dilgen et al. (2013) and Oprisan et al. (2015). Briefly, male PV-Cre mice (B6; 129P2 – Pval^*btm*1(*Cre*)*Arbr*/*J*^ Jackson Laboratory (Bar Harbor, ME, USA) were infected with the viral vector (AAV2/5. EF1a. DIO. hChR2(H134R) – EYFP. WPRE. hGH, Penn Vector Core, University of Pennsylvania) delivered to the mPFC as described in detail in Dilgen et al. (2013). The extracellular signals were amplified using a Grass amplifier (Grass Technologies, West Warwick, RI, USA), digitized at 10 kHz by a 1401plus data acquisition system, visualized using Spike2 software (Cambridge Electronic Design, LTD., Cambridge, UK) and stored on a PC for offline analysis. A HumBug 50/60 Hz Noise Eliminator (Quest Scientific Inc., Canada) filter canceled out the line noise and the signal was band-pass filtered online between 0.1 and 130 kHz to obtain the LFPs. A 473 nm laser (DPSS Laser System, OEM Laser Systems Inc., East Lansing, MI, USA) delivered the light stimulation via a 1401plus digitizer and Spike2 software (Cambridge Electronic Design Ltd., Cambridge, UK).

## 3. Data analysis

As in the first paper of this series (Oprisan et al., 2015), for each 2 s long LFP recording in response to a brief 10 ms light pulse, the first approximately 0.5 s were discarded to remove the transient response of the neural network and only analyze the last 1.5 s of steady oscillatory activity of the network. The transient response is dominated by the transient phase resetting in response to a brief 10 ms optical stimulation (see Oprisan et al., 2015 for a detailed procedure of measuring network-level phase resetting). While phase resetting at the neural network level can provide invaluable information regarding the ability of stimuli to drive the network with meaningful applications in, for example, epilepsy (Osorio and Frei, 2009; Parastarfeizabadi and Kouzani, 2017) or Parkinson's (Tass, 2000, 2003), here we focused on the steady state, unperturbed, activity of the network. The goals of this study were to (1) identify possible low-dimensional stable attractors of neural activity during the steady activity of the network, and (2) compare neural activity under systemic cocaine against the control data published in Oprisan et al. (2015).

### 3.1. Nonlinearity tests

Without repeating all the mathematical details from Oprisan et al. (2015), we again tested for nonlinearity in cocaine-induced changes in neural activity. The nonlinearity tests are necessary since some algorithms, e.g., for computing the embedding dimension of a time series, give similar results both for linear stochastic processes and for deterministic data (Osborne and Provencale, 1989). To distinguish nonlinearity from purely stochastic time series (Osborne and Provencale, 1989; Small et al., 2001), we used surrogate data (Theiler et al., 1992; Small, 2005) with the null hypothesis that the data is linearly correlated in the temporal domain, but are random otherwise (Cogranne and Retraint, 2013). The surrogate data were generated by randomizing the Fourier transform phases, which is known to preserve the linear correlations within the original data while destroying any nonlinear structure (Theiler et al., 1992; Garcia et al., 2013).

The rate of false rejections of the null hypothesis determines the necessary number of surrogates to be generated (Jung et al., 2003). At least *n* = 1/*l* surrogates should be generated to attain a certain level *l* of significance, e.g., for a significance level of *l* = 0.05 at least *n* = 1/*l* = 20 surrogates are required (Jung et al., 2003; Yuan et al., 2004). In general, a set of values λ_*i*_ (with *i* = 1, …, *n*) of the discriminating statistics is computed for surrogates and compared against the value λ_0_ for the original time series. Rejecting the null hypothesis can be done using rank ordering, in which case λ_0_ must occur either on the first or on the last place in the ordered list of all values of the discriminating statistics to reject the null hypothesis. Alternatively, the null hypothesis could be rejected using the average statistical method, in which case a score γ is derived as follows:

$$\gamma =|\frac{\bar{\lambda }}{\lambda_{0}}-1|,$$

where $\bar{\lambda }=\frac{1}{n}\overset{n}{\underset{i=1}{\sum }}\lambda_{i}$ is the mean value of the discriminating statistics over all surrogates. If γ > 1, then the original data and the surrogates are significantly different and the null hypothesis is rejected (Yuan et al., 2004). Finally, the null hypothesis could be rejected using the coefficient of variation statistical method, in which case a score γ is derived as follows:

(1) $$\begin{array}{r} \gamma =|\frac{\bar{\lambda }-\lambda_{0}}{\sigma_{\lambda }}|, \end{array}$$

where σ_λ_ is the standard deviation of the discriminating statistics over all surrogates. Assuming a normal distribution for λ_*i*_, rejection of the null hypothesis requires γ > 1.96 at a 95% confidence level (Stam et al., 1998; Jung et al., 2003).

We used the time reversal asymmetry method (Costa et al., 2005) for surrogate data to compute both the individual trial measure, λ, and the cumulative statistical scores, γ, for both (1) the average statistics (Jung et al., 2003; Yuan et al., 2004) and (2) the coefficient of variation of the distributions of λ's (Theiler et al., 1992; Kugiumtzis, 2002; Jung et al., 2003). As before (Oprisan et al., 2015), for every trial and every animal we generated 100 surrogates and, in addition to the γ scores (Stam et al., 1998; Jung et al., 2003), we used the percentage of false nearest neighbors to check the time series nonlinearity (Birkelund et al., 2004; Kugiumtzis and Tsimpiris, 2010). Although our time series were long enough and recorded with a high sampling rate, we also explored additional null hypothesis testing methods used for testing very short time series (Caillec and Montagner, 2013).

A time series is said to be reversible if its probabilistic properties are invariant with respect to time reversal (Diks et al., 1995). For example, a simple Gaussian random walk is time-reversal invariant (Weiss, 1975). Practical implementations of temporal asymmetry measures use, for example, the difference between the probability density functions of the original and time-reversed series, or of their corresponding variances (Zumbach, 2009, 2012), or temporal-based correlation measures over different temporal windows, or between the past and future data blocks of the same temporal length (Zamparo et al., 2013), or by using Granger causality (Winkler et al., 2016). Time irreversibility is a strong signature of nonlinearity (Schreiber and Schmitz, 2000) and is commonly related to entropy production by the underlying (often unknown) mechanism that generated the time series (see Vladimirov and Petersen, 2010; Roldán, 2014 and references therein). As in the previous study (Oprisan et al., 2015), the null hypothesis assumes that the time series is produced by a linear Gaussian random process (Diks et al., 1995). We used the Tisean software package to compute the time reversal asymmetry statistics both for the original and the surrogate data (Hegger et al., 1999; Schreiber and Schmitz, 2000; Oprisan et al., 2015). Given that (1) our time series did not require a significant computational overhead by generating the surrogates, and (2) we already know the time scale of the processes we are interested in capturing, we did not consider additional nonlinearity tests, such as the horizontal visibility algorithm and the Kullback-Leibler divergence (Lacasa et al., 2012). When using the rank ordered statistics for time reversal asymmetry (see Oprisan et al., 2015 for detailed definitions), the original data had a value of λ_0_ = 0.2, and the surrogate data had a significantly different value of λ = 2.5, which rejects the null hypothesis. Additionally, the γ score of the coefficient of variation statistics was above the 1.96 threshold, and therefore we rejected the null hypothesis (see Oprisan et al., 2015 for a detailed mathematical description of the statistical tests). As a result, we concluded that the above statistical tests support the hypothesis of a nonlinear structure in our data.

### 3.2. Phase resetting of LFP

**Figure 1:**
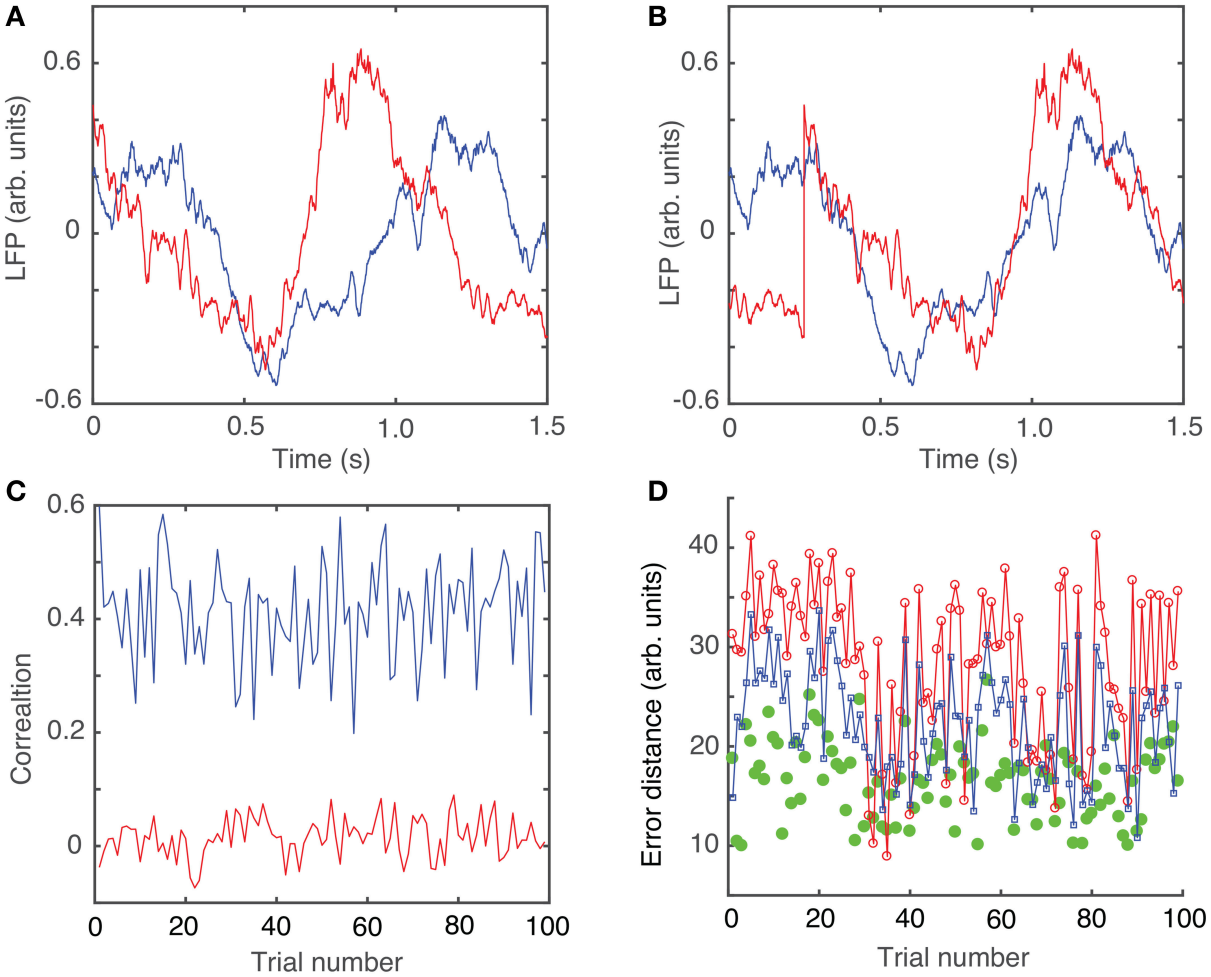
Phase resetting of neural network activity from LFPs. Steady LFP activity between a “reference” trial (blue) and another arbitrary trial **(A)** without phase shift and **(B)** with a circular phase shift that maximizes the coefficient of correlation. **(C)** Without phase shifting to correct for the phase resetting, the average correlation was 0.025 ± 0.035 (red trace), which increased after appropriately phase shifting to 0.414 ± 0.089 (blue trace). **(D)** The root-mean-square (*rms*) error between the two trials decreases from 28.3 ± 7.8 without phase resetting correction (red line), to 22.3 ± 5.5 after phase-shifting (blue line), to 16.6 ± 3.8 for phase-shifted dendrogram-based correlation (green solid circles).

External perturbations, such as synaptic inputs, light, or mechanical pressure, alter the ongoing rhythm of oscillators by changing both their phases and amplitudes (Oprisan and Canavier, 2002; Oprisan et al., 2004; Oprisan, 2013; Oprisan and Austin, 2017). Brief perturbations applied to intrinsic oscillatory activities lead only to transient changes of the rhythm, which eventually dissipate after a few cycles. Since our focus is on identifying similarities among steady state LFPs, any transient changes in the phases of LFP oscillations should be removed (Oprisan, 2017). As previously described (Oprisan, 2017; Oprisan and Austin, 2017), the phase resetting was estimated by the amount of required circular shift on each LFP trace (Figure 1A) in order to maximize the coefficient of correlation between any trial and an arbitrary selected “reference” trial (Figure 1B). Phase resetting correction led to a significant increase in the coefficient of correlation from 0.025 ± 0.035 (red trace in Figure 1C) to 0.414 ± 0.089 (blue trace in Figure 1C). Additionally, the root-mean-square (*rms*) error, i.e., the Euclidian norm of the difference between each 1.5 s long trial and the arbitrary “reference” trial, decreased from 22.3 ± 5.5 (red line in Figure 1D) to 16.6 ± 3.8 (blue line in Figure 1D).

### 3.3. Dendrograms of phase shifted LFPs

A dendrogram is a visual representation of “relationships” among trials. A possible quantitative measure of the “relationship” is through the correlation coefficient (Saraçli et al., 2013), although in this study we used the Euclidian distance as implemented in Matlab (Hill and Lewicki, 2005). The trials are aligned along the horizontal direction of the dendrogram plot and are called “leaf” nodes, whereas the vertical axis is an appropriately-defined “distance.” For example, if the correlation coefficient (*c*) is the measure of trials' similarity, then the distance between two trials is *d* = 1 − *c*, i.e., higher the correlation coefficient smaller the distance between “leaf” nodes (trials).

**Figure 2:**
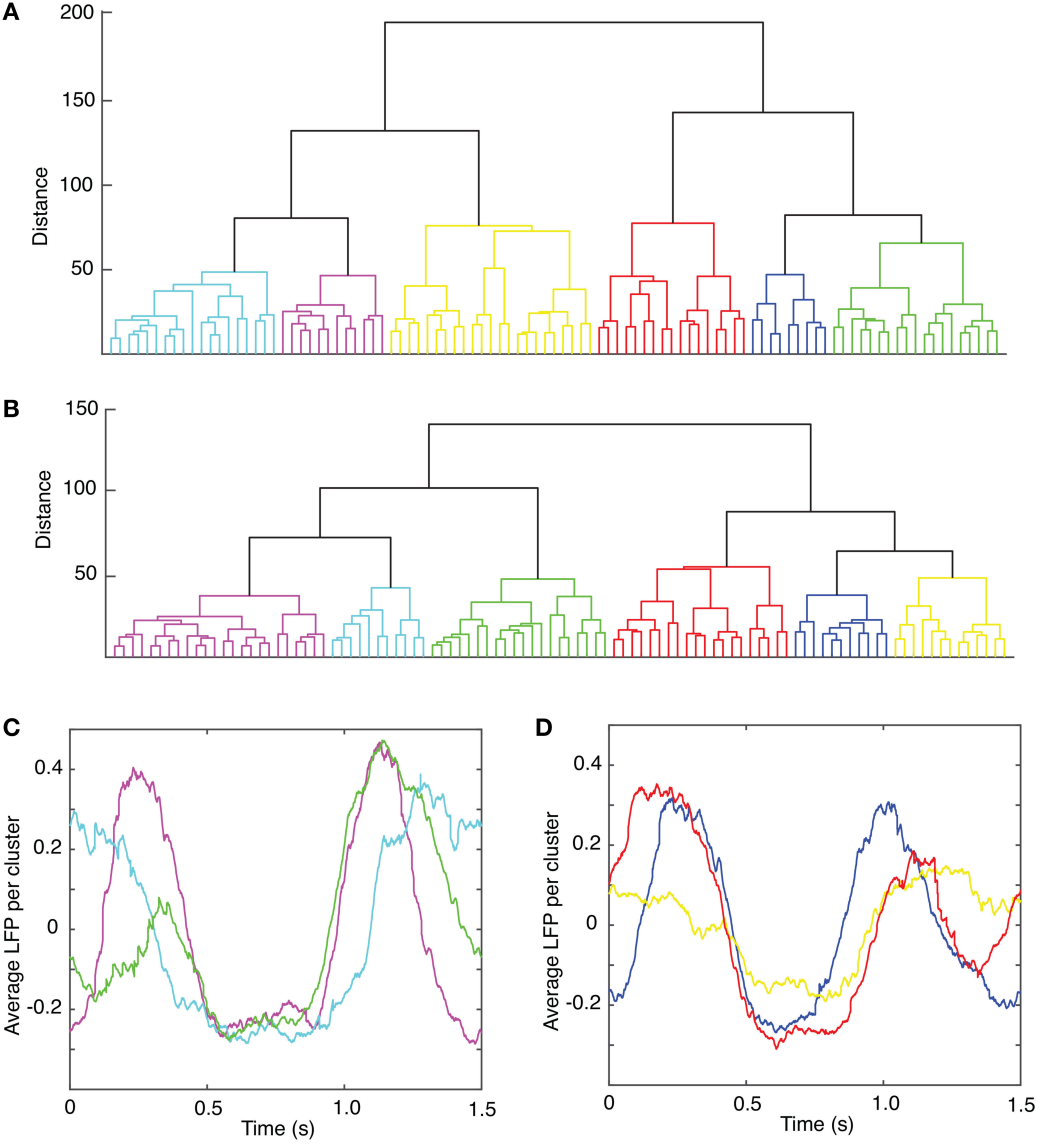
Dendrogram-based grouping of similar LFPs. Six similar clusters are formed out of the 100 trials both without **(A)** and with **(B)** trial phase shifts to account for network phase resetting. The distance threshold that generates six clusters is about 80 units without phase correction **(A)** and decreases to about 60 units **(B)** after phase correction. The corresponding average LFPs for each of the clusters of the optimized dendrogram show large **(C)** and smaller **(D)** amplitude oscillations.

The circular shift, performed in the previous section with the purpose of maximizing the coefficient of correlation between any trial and an arbitrary “reference” trial, helps defining the relative phase of the trials with respect to each other. The hierarchical classification of trials in dendrogram groups suggests “similar”-looking clusters of trials that may have a similar mathematical description. The *rms* error computed between each trial and its corresponding cluster average further decreases (see green solid circles in Figure 1D), suggesting a strong correlation among the clustered trials. Throughout this study, we only used the Euclidian distance to measure similarities among the “leaf” nodes of the dendrogram (see Figure 2A). As we noticed, phase resetting correction significantly lowered the distance among similar trials. For example, the distance threshold for breaking the trials in six clusters before correcting for phase resetting was about 80 units (see Figure 2A) and after phase correction was about 60 units (see Figure 2B). In Figures 2C,D we plotted the average trace of each cluster from the optimized dendrogram shown in Figure 2B. There are some clear differences between the cluster traces, e.g., clusters 1 (magenta trace in Figure 2C) and 2 (green trace in Figure 2C) have high amplitude oscillations whereas cluster 6 (yellow trace in Figure 2D) has a very small peak-to-peak amplitude. Although we only show figures for one of the six animals, the same numerical procedure was applied to all the data.

## 4. Delay embedding method

Membrane potential oscillations are generated by intricate feedback mechanisms that involve many different types of ionic channels (Hille, 2001). Although in the simplest possible conductance-based model only a fast sodium and a potassium delayed rectifier suffice (Hodgkin and Huxley, 1952), more accurate models have hundreds of compartments each populated with tens of different ionic channels (Buzsáki and Draguhn, 2004; Schnitzler and Gross, 2005). Even the simplest conductance-based model requires evolution equations for four independent variables (membrane potential, activation variable for both sodium and potassium and inactivation of potassium channels) (Hodgkin and Huxley, 1952). The number of independent variables required for a model is its dimensionality. For pyramidal cells realistically interconnected to mimic the mPFC network, we would expect an extremely large number of dimensions (Schnitzler and Gross, 2005). However, when the actual model equations are not known, finding the dimensionality of a nonlinear system falls on experimental data. Nonlinear dynamics (Abarbanel, 1996; Kantz and Schreiber, 1997; Schuster and Just, 2005) developed a set of tools for data mining, which include phase space embedding. In this paper series (see also Oprisan et al., 2015), we investigated the LFPs using delay-embedding method of nonlinear dynamics to estimate the number of degrees of freedom of the steady activity of mPFC neural network.

One of the challenges of delay-embedding is that we only record one-dimensional data (time series) of the membrane potential and use it to identify all the other independent variables that describe the system. The delay embedding method (Packard et al., 1980; Takens, 1981), takes a time series *x*_*i*_ = *x*(*i*Δ*t*) with *i* = 1, 2, …, *N* where *N* is the number of data points and Δ*t* is the uniform sampling time, and expands it into a *d*−dimensional vector:

$$x_{i}=\left(x_{i},x_{i+n},\ldots ,x_{i+\left(d-1\right)n}\right),$$

where τ = *n*Δ*t* is the delay, or lag, time.

### 4.1. The lag time

One potential problem with selecting the “right” delay time is that a too small value leads to highly correlated embedded vectors. Geometrically, all the data points cluster along the diagonal direction of the embedding space leading to a one-dimensional attractor, regardless the complexity of the original data. This issue is known as *redundancy*, and the obvious solution is to increase the delay time τ until the components of the embedded vectors become independent (Casdagli et al., 1991). However, the delay time cannot be arbitrarily large because then the reconstructed vectors are completely de-correlated. Geometrically, the data points will uniformly fill out the entire phase space without showing any particular structure (Casdagli et al., 1991). As in the previous study (Oprisan et al., 2015), we used both (1) the autocorrelation (Holzfuss and Mayer-Kress, 1986; King et al., 1987; Zeng et al., 1991; Schiff and Chang, 1992; Schuster and Just, 2005) and (2) the average mutual information (AMI) (Fraser and Swinney, 1986; Kantz and Schreiber, 1997; Hegger et al., 1999) for estimating the lag time τ.

### 4.2. The embedding dimension

As before (Oprisan et al., 2015), Takens' theorem (Takens, 1981) provided a rough estimate of the embedding dimension through its practical implementation in the false nearest neighbors (FNN) algorithm (Kennel et al., 1992; Hegger et al., 1999; Sen et al., 2007). The intuitive idea behind FNN is that high-dimensional phase space trajectories projected onto a lower dimensional embedding space will show self-crossing points. Such false crossing points could be eliminated by unfolding the attractor in the right dimensional space (Kennel et al., 1992).

## 5. Reconstructed neural activity under cocaine

### 5.1. Experimental data

Every 1.5 s-long LFP trial was first circularly shifted to correct for the phase resetting induced by the brief 10 ms light stimulus (see Figure 1B).

**Figure 3:**
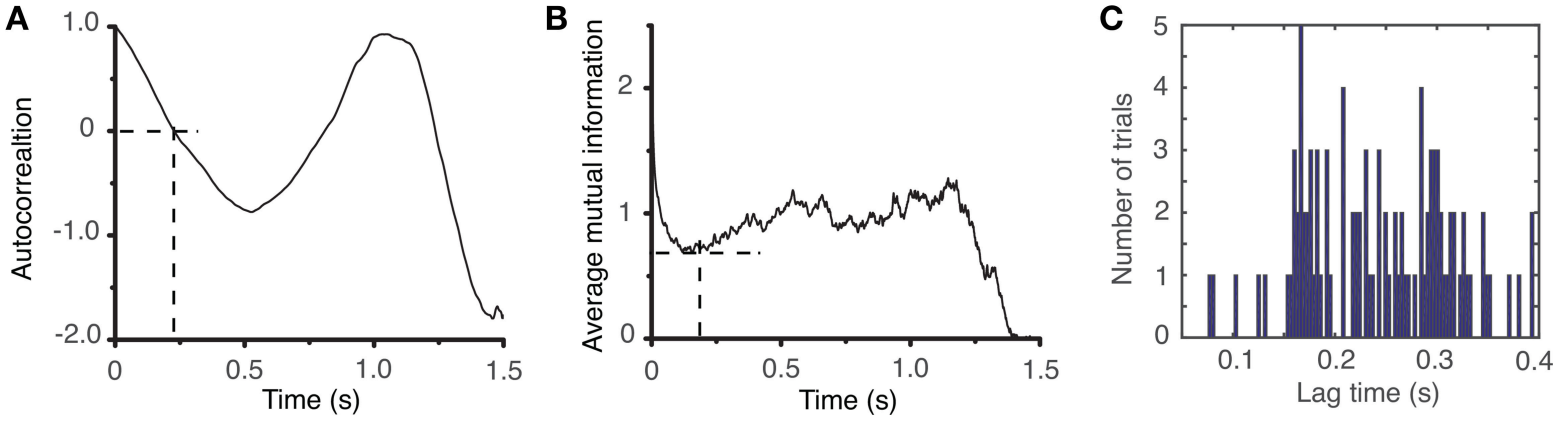
Time lag estimation. The first zero crossing of the autocorrelation function is around τ ≈ 2,400Δ*t* **(A)** and the first minimum of the average mutual information is around τ ≈ 2,300Δ*t* **(B)** with Δ*t* = 10^−4^s. The histogram of all lag times for animal #1 has a broad range with a mean of about τ_*mean*_ = (2,428 ± 707)Δ*t* ≈ (0.24 ± 0.07) s **(C)**.

The lag time was estimated both with (1) the autocorrelation (Casdagli et al., 1991), and (2) the average mutual information method (Fraser and Swinney, 1986). The autocorrelation measures the amount of linear correlation between the time series and a time-shifted version of itself. By selecting for the lag time the first zero crossing of the autocorrelation (see Figure 3A), we ensure that any (linear) correlation between the two time series was removed (Abarbanel, 1996). Since the autocorrelation method only eliminates the linear correlation between a time series and its time-shifted version, we also used the first minimum of the nonlinear autocorrelation function called *Average Mutual Information* (AMI) (Fraser and Swinney, 1986; Kantz and Schreiber, 1997) to estimate the lag time (see Figure 3B). The distribution of all autocorrelation-based lag times for animal #1 is shown in Figure 3C and the corresponding dendrogram-based cluster averages are given in Table 1. Although only the autocorrelation-based lag times are shown both in Figure 3C and Table 1, the AMI-based lag time values (not shown) were within 5% of those obtained with the autocorrelation method. As before (Oprisan et al., 2015), we used the Tisean function *autocor* to compute the autocorrelation (see Figure 3A) and *mutual* to compute the AMI (Figure 3B).

**Table 1:** Estimated average lag times for each cluster of the dendrogram.

**Mouse #**	**Cluster 1**	**Cluster 2**	**Cluster 3**	**Cluster 4**	**Cluster 5**	**Cluster 6**
1	1,724	1,810	2,966	3,114	2,589	2,857
2	4,038	3,982	2,009	2,530	4,548	932
3	2,924	2,104	943	3,797	1,654	3,316
4	1,114	5,417	1,347	1,345	1,688	2,540
5	4,901	4,225	4,079	1,096	4,022	5,000
6	5,453	4,779	2,902	3,415	1,859	3,156

### 5.2. Embedding dimension

**Figure 4:**
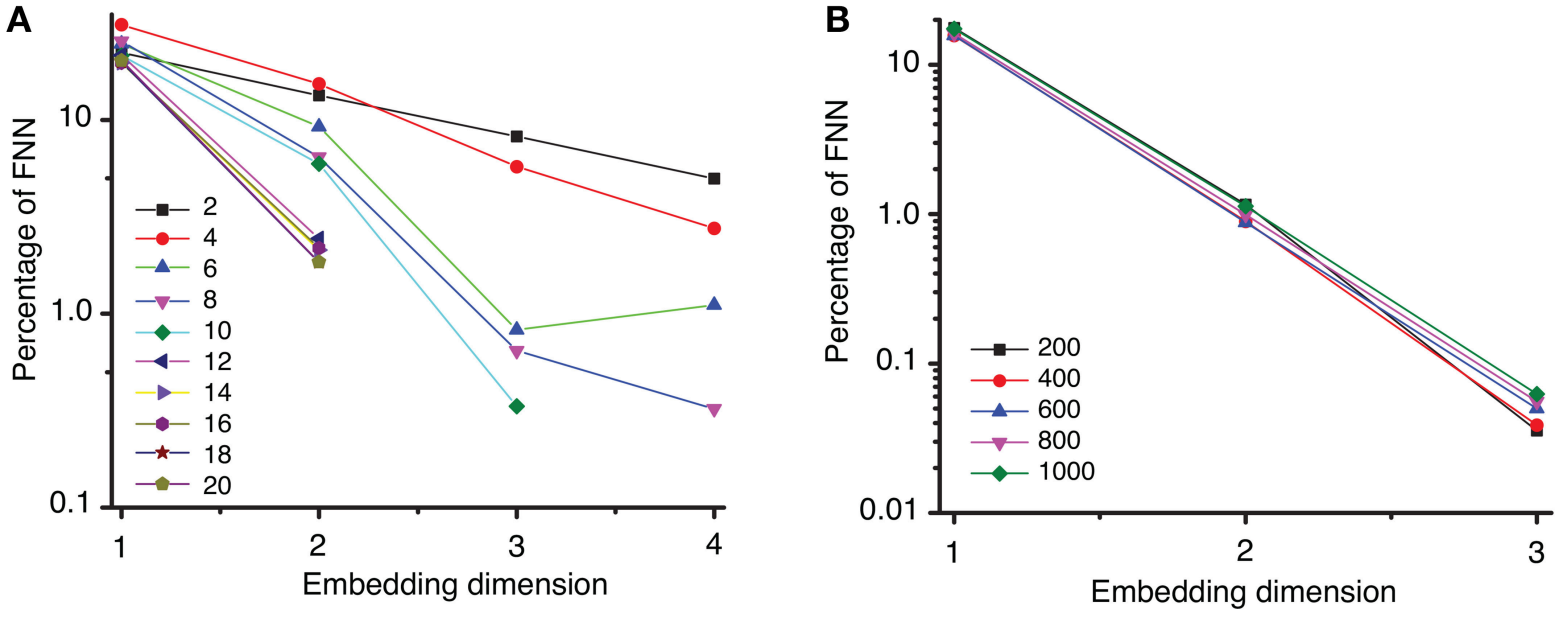
Percentage of false nearest neighbors. **(A)** A semi-log plot of the percentage of FNN shows that for a distance ratio *f* > 7 the percentage drops below 1% for an embedding dimension of *d*_*E*_ = 3. This suggests that an optimum ratio is above *f* = 7, in agreement with results from others (Abarbanel, 1996; Konstantinou, 2002). **(B)** Spurious temporal correlations among too closely spaced data points were removed by different Theiler windows from 100 to 8,000 samples, i.e., from 0.01 to 0.8 s (only a small subgroup is shown here). The percentage of FNN drops below 0.1% for an embedding dimension of *d*_*E*_ = 3.

Once we obtained a consistent estimate of the lag time, the Tisean package was used for computing the embedding dimension of the data (see Oprisan et al., 2015 for explicit Tisean function calls). In the examples shown in Figure 4A, we used a lag time τ_*mean*_ = 2428Δ*t* to estimate the embedding dimension with variable distance ratios, *f*, between 2 and 20. The distance ratio *f* is given by the distance between two points in a (*d* + 1)-dimensional space relative to its value in the *d*-dimensional space. Distance ratio (Kennel et al., 1992; Abarbanel, 1996) is sometimes called “the escape factor” (Kugiumtzis, 2002) since by increasing the embedding dimension from *d* to (*d* + 1) false neighbors move quickly apart. Too small of a distance ratio leads to an overestimation of the percentage of false neighbors, whereas too large of a distance ratio gives a large number of false positives. For example, for large distance ratios, e.g., *f* > 7, the percentage of false nearest neighbors drops below 1% for an embedding dimension *d*_*E*_ = 3 (see Figure 4A). For all values of the distance ratio *f* > 12, the percentage of FNN dropped below 10^−6^% at embedding dimension *d*_*E*_ = 3 (not shown in Figure 4A).

Strong temporal correlations are expected among data points that are close to each other (Theiler, 1990; Theiler et al., 1992). As a result, such data points should be removed, i.e., the time series must be “windowed” to avoid spurious temporal correlations (Grassberger, 1987; Theiler, 1990). Among the most used Theiler window criteria is three times the correlation time (Heath, 2000), (*d* − 1)τ, or the space-time separation distance (Provenzale et al., 1992). We tested a wide range of Theiler windows from 100 to 8,000 sampling times, which allowed us to account for any possible strong temporal correlation of closely spaced data points. For all Theiler windows tested, the percentage of FNN dropped below 0.1% at embedding dimension of *d*_*E*_ = 3 (see Figure 4B). Although we tested a wide range of Theiler windows, we only show five representative results in Figure 4B for two reasons: (1) the trend in the omitted data is similar and does not add anything to the data shown in Figure 4B, and (2) avoid figure cluttering.

**Figure 5:**
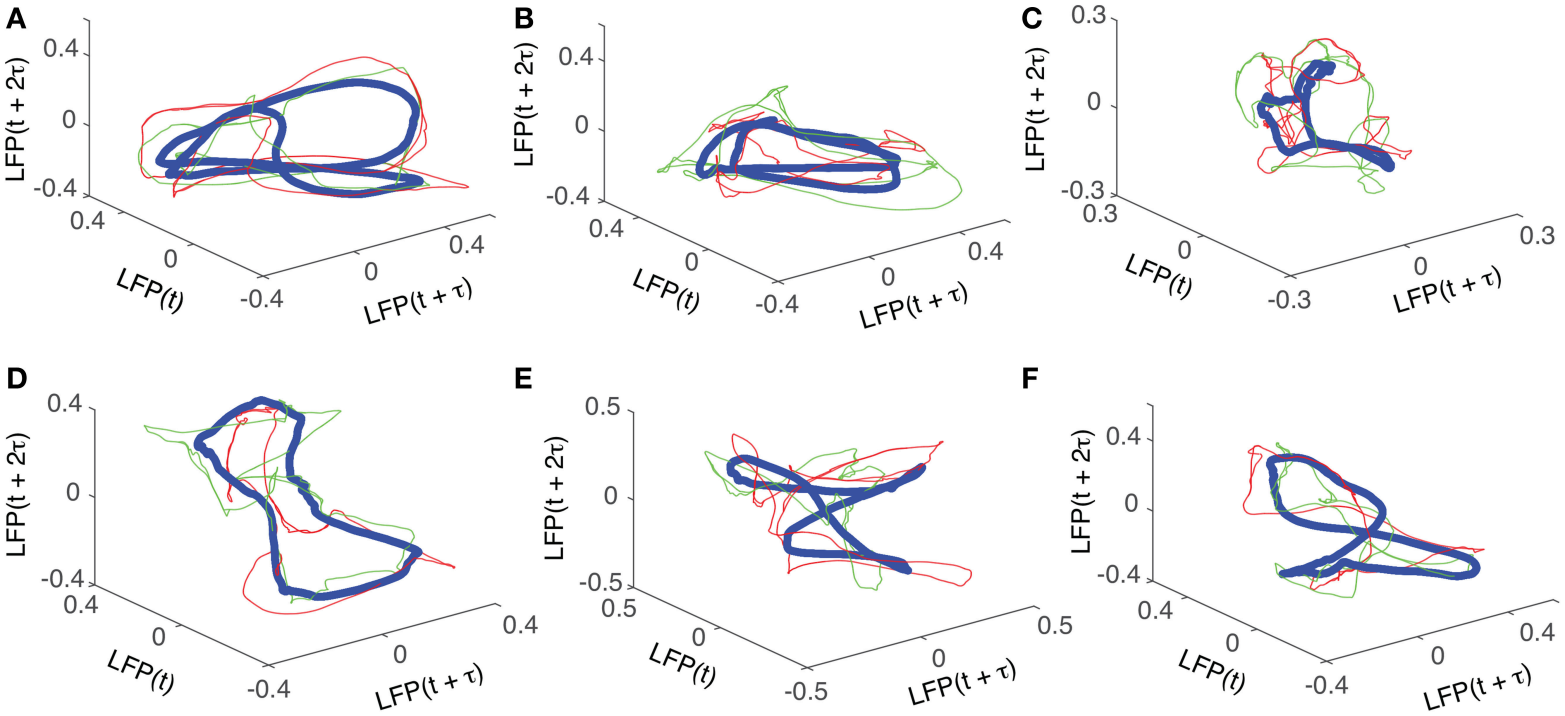
Reconstructed 3-dimensional attractors. For each of the six dendrogram-based clusters (see Figure 2B) two randomly selected trials (red and green thin lines) together with the cluster average (blue thick line) show the reconstructed attractors. The cluster average (blue thick line) only serves as a visual aid to guide us gauge if the two randomly selected trials from the same cluster remain close to each other al all times.

For each trial, the phase space attractor was reconstructed using its corresponding delay (lag) time, of which we only show two examples from each cluster, represented by the red and green thin lines in Figure 5. For each cluster, we also show the average reconstructed trace with the thick blue line in Figure 5. Although the reconstructed cluster average (blue thick traces in Figure 5) does not represent any “true” attractor, we used it here mostly as a visual aid that helps us gauge how individual phase space trajectories relate to the average. Although it seems as if all the attractors are different, they are topologically equivalent (see Oprisan et al., 2015), i.e., by changing the delay time one phase space trajectory could be morphed into the other. The attractors in Figures 5A,B show a double loop (period-2 attractor), which can be “unfolded” into the “8”-shape in Figures 5E,F by slightly changing the delay time. Furthermore, any “8”-shaped attractor is topologically equivalent with a closed elliptic attractor as shown in Figures 5C,D. Indeed, the attractor in Figure 5D could be morphed into the one shown in Figure 5F by twisting the upper half of the attractor with respect to the lower half. Moreover, an “8”-shaped attractor such as the one shown in Figure 5F could be morphed into a period-2 attractor as in Figure 5B by folding the front two loops.

The detailed procedure described above was also applied to the other five data sets from the different animals (not shown). In summary, the delay embedding method gave consistent delay (lag) time estimations with both the autocorrelation and the average mutual information methods (see Table 1) and the optimum delay embedding dimension was *d*_*E*_ = 3.

## 6. Control vs. cocaine neural activity

What did we learn from analyzing the neural activity under cocaine? First, we found that the delay embedding method also works for the cocaine case as well (see Oprisan et al., 2015 for a discussion of control data). Second, we found that the embedding dimension is still three, the same as for the control data in Oprisan et al. (2015). This means that the mathematical model that could describe both the control and the cocaine cases only require three independent variables. This is significant because it opens the possibility of deriving a single mathematical model, possibly with only (slightly) different control parameters to describe both control and cocaine results.

Are control data truly different in a significant way when compared against cocaine to warrant such a hypothesis? To answer this question, we compared the data side-by-side from the same animals before and after cocaine using a dendrogram similarity measure. For each of the six animals, we concatenated the 100 trials before cocaine (trial index from 1 to 100) with the 100 trials after cocaine (trial index 101 to 200) in a single 200-trial file. As it was described above, after performing appropriate phase shifting to account for the transient phase resetting induced by the light stimulus (see also Oprisan et al., 2015), we performed a dendrogram analysis. If the neural activity patterns before and after cocaine are totally distinct, then we would expect that they separate in clusters with no overlap. A very high degree of similarity would be troubling from the modeling point of view since it would indicate that we cannot distinguish between the two conditions using this dendrogram method.

**Figure 6:**
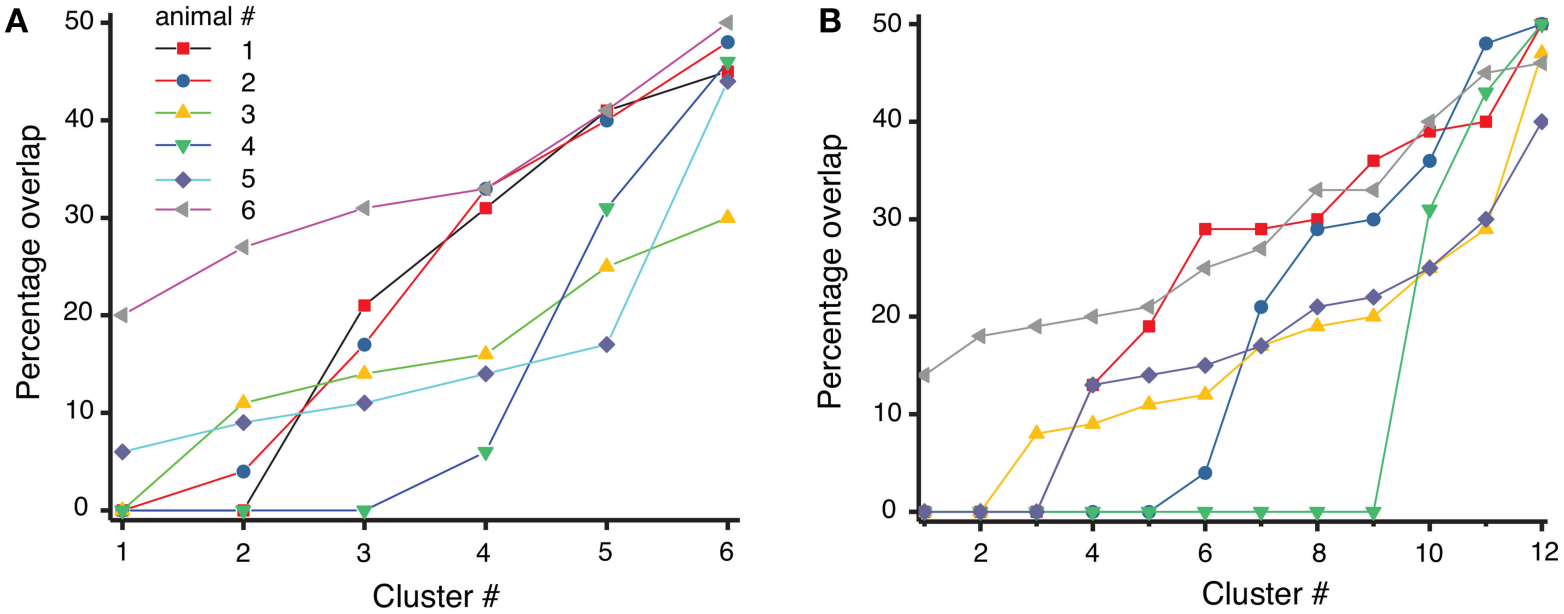
Overlap index between before and after cocaine for each of the six animals. The overlap index varies from zero (total separation of before from after cocaine trials) to maximum possible overlap where 50% of trials are from before and the other 50% are from after cocaine in the same cluster. The overlap seem to be consistent regardless the number of clusters: in six-cluster dendrogram **(A)** the mean percentage overlap is (21.2 ± 1.1)%, whereas in 12-cluster dendrogram of the same data the mean overlap is (18.9 ± 1.1)% **(B)**.

For each cluster, we computed the percentage of mixing between before and after cocaine trials by using the formula:

(2) $$\begin{array}{r} \%\;overlap=\frac{min\left(\#trials_{before},\#trials_{after}\right)}{\#trials_{cluster}}, \end{array}$$

where *#trials*_*cluster*_ represents the total number of trials in a given cluster, of which *#trials*_*before*_ belong to the experiments before cocaine injection (trial index 1 to 100) and *#trials*_*after*_ belong to the experiments after cocaine injection (trial index 101 to 200). If a dendrogram cluster contains only one category of trials (either only before or only after cocaine trials), then the overlap is zero and the separation between before and after cocaine trials is maximum possible. However, if half of the trials of a cluster are from before and the other half are from after cocaine, then the overlap is maximum possible and the dendrogram cannot discriminate between those trials. We used both the six-cluster (see Figure 6A) and the 12-cluster (see Figure 6B) dendrograms to classify the 200 combined trials. The difference in the average percentage of trial overlap between the two cases is not significant, i.e., (21.2 ± 1.1)% for six-cluster and (18.9 ± 1.1)% for 12-cluster dendrograms. The fact that the average percentage overlap does not change with the number of clusters of the dendrogram suggests that the amount of overlap could be determined by a true similarity among the three-dimensional reconstructed dynamics both before cocaine (see Oprisan et al., 2015) and after cocaine (as in this work). As a result, it may be possible to fit the same three dimensional mathematical model to the data before cocaine with one set of model parameters and the after cocaine data with the same model but with a different set of parameters. The almost 20% average mix between before and after cocaine could be the result of a small set of parameters that remain constant between the two models, i.e., the most stable, or invariant, part of the model.

**Figure 7:**
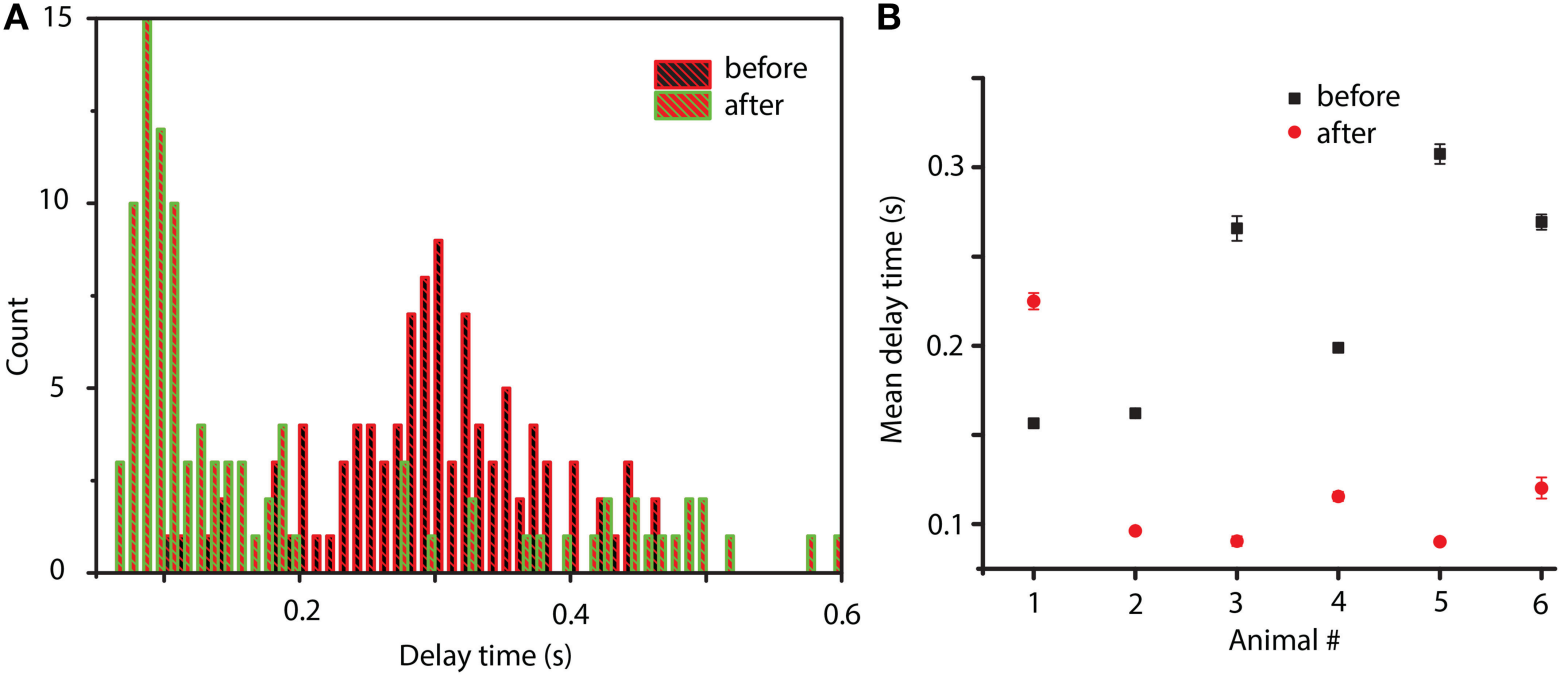
Distribution of delay times before and after cocaine. Typical delay time distributions show a clear time scale separation between conditions: after cocaine the delay times are significantly smaller than before cocaine **(A)**. The average value of the lognormal fit for the six animals show that for all animals except one, the after cocaine mean delay time is shorter (two to three times shorter) than for before cocaine **(B)**.

We also know that between before and after cocaine, there are significant differences that a possible mathematical model must capture (besides the common or invariant part represented by a 20% similarity among phase space reconstructions). Indeed, from a dynamical point of view, we found significant differences between before and after cocaine trials. For example, the delay times for the phase space reconstructions are significantly different (see Figure 7). Although for each trial the optimal delay is different, it is clear that the distributions of delay times are also significantly different between before and after cocaine (see Figure 7A). We notice that, on average, all delay times are larger for before cocaine trials compared to after cocaine (see Figure 7A). We fitted the distributions with lognormal functions to capture correctly the long tail of distributions and showed that the center of the delay time distributions before and after cocaine are well separated (see Figure 7B). In all animals except one, the centers of lognormal delay time distributions for before cocaine trials are two to three times longer (see Figure 7B) than for cocaine trials. This has a significant impact on the mathematical modeling since the delay time sets the time scale of model equations. One possible approach to modeling such a difference in delay times between control and cocaine trials could be along the line of inquiry of previous experiments done by Dilgen et al. (2013), who speculated that cocaine may enhance synchronization of neural activity via a tighter PV-cell induced oscillation.

**Figure 8:**
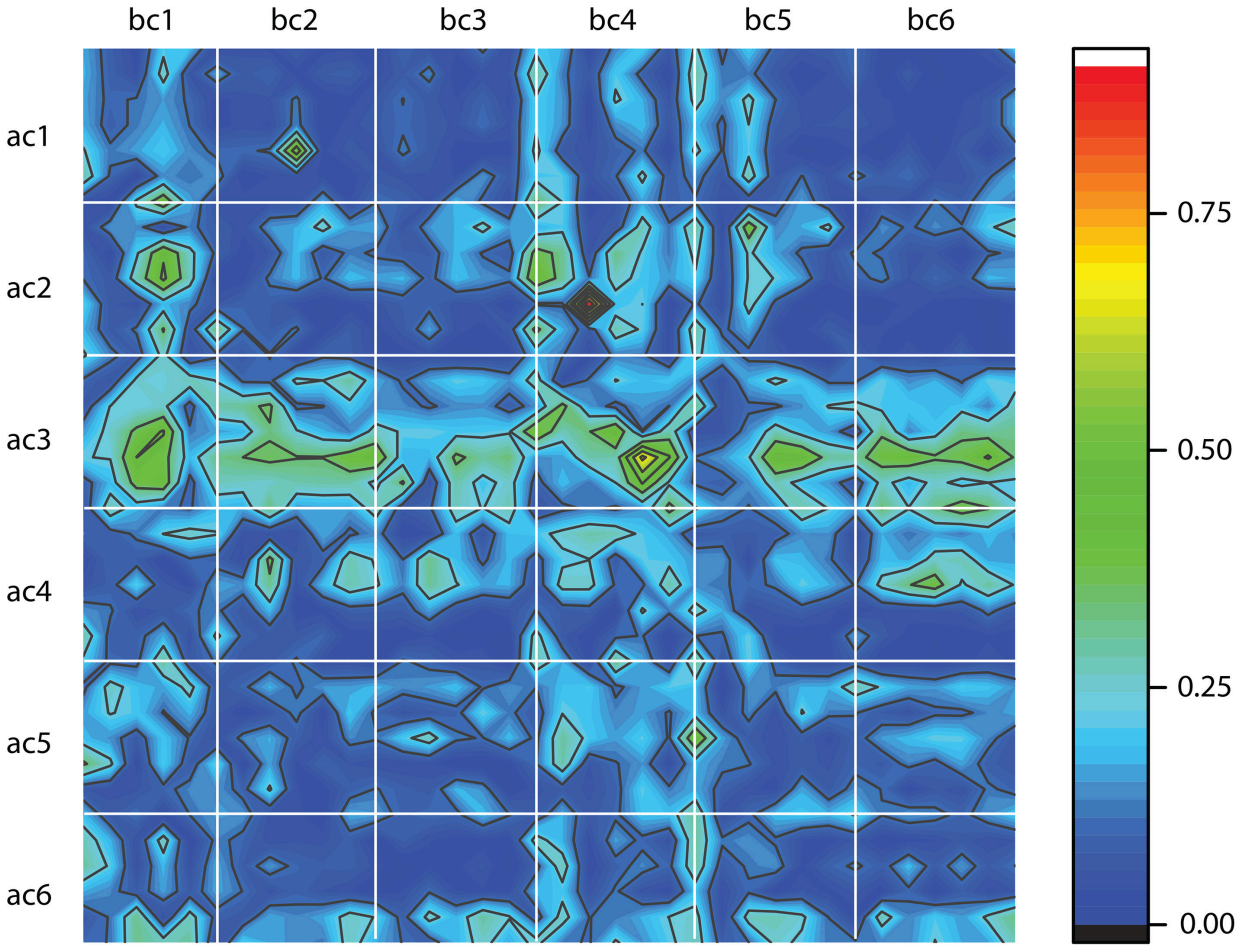
Frechet distance between the control, before cocaine (bc) and after cocaine (ac) trials for all six animals. Each white-bordered square contains the color-coded Frechet distance between the 100 control (bc) trials and the 100 after cocaine (ac) trials. Deep blue colors mean small Frechet distance and suggests more similar phase space trajectories.

The above classification method is entirely based on dendrogram's Euclidian distance between phase shifted trials. However, the one-dimensional time series do not tell the entire story of neural activity. Therefore, after we reconstructed the three-dimensional attractors using the delay-embedding method, we computed the Frechet distance between phase space reconstructed trajectories (Frechet, 1906). Frechet distance can be intuitively formulated in terms of a man walking a dog on a leash. The man walks along one curve whereas the dog along the other and Frechet distance is the shortest leash that is sufficient for traversing both curves (Eiter and Mannila, 1994). In other words, the Frechet distance is a measure of the similarity between two curves in any metric space by taking into account the location and ordering of the points along the curves (Eiter and Mannila, 1994). We used a readily available Matlab implementation of Frechet distance algorithm (Danziger, 2013) and computed all distances between any possible combinations of the 100 trials before cocaine (bc) with the 100 trials after cocaine (ac) for all six animals. In Figure 8, each white-bordered square corresponds to a combination of 100 × 100 trials. For example, the top left square shows the color-coded Frechet distance between the 100 trials before cocaine for the first animal (labeled bc1) and the 100 trials after cocaine for the first animal (labeled ac1). Deep blue colors indicate small Frechet distances, i.e., more similar reconstructed attractors. We must emphasize that this is a different kind of similarity measure compared to the dendrogram similarity described above. This is because the Frechet distance was computed between the three-dimensional reconstructed attractors rather than the one-dimensional LFPs. As a result, Frechet distance includes dynamic information regarding both the lag time and the embedding dimension of the reconstructed attractors. A cursory inspection of Figure 8 suggests some patterns. For example, all after cocaine trials for animal #3 (ac3) have large Frechet distances to any control trial across all animals (bc1 to bc6). Similarly, it seems that the control data for animal #4 (bc4) have consistent large Frechet distances with respect to all after cocaine trials across all animals (ac1 to ac6). The dynamic information contained in the Frechet distance plots shown in Figure 8 could potentially provide additional hints regarding the time scales of variables involved in a future modeling of LFPs. A consistent blue color due to a small Frechet distance between trials suggests very similar phase space dynamics, i.e., possibly with very close time scales.

## 7. Discussion

As in the previous study (Oprisan et al., 2015), we performed both a phase shifting on the LFPs to correct for the phase resetting induced by light stimulus, and a grouping of the shifted LFPs in similar patterns of activity using a dendrogram (see Figure 2).

We successfully reconstructed three dimensional attractors based on LFPs from mPFC of ChR2 expressing PV+ interneurons. The delay time for each trial was estimated using both the autocorrelation and the average mutual information functions (see Table 1). We used the false nearest neighbor method and found that the minimum embedding dimension was *d*_*E*_ = 3 for all six animals.

We found topologically similar phase space attractors that could be morphed into each other through an appropriate change in delay time (see Oprisan et al., 2015 for details). The characteristic “8”-shaped attractor (see Figure 5) was also found in the control cases (Oprisan et al., 2015).

Both the study on control data (Oprisan et al., 2015) and this study on cocaine modulated neural activity suggest that the local network could be described by a model with only three variables. Furthermore, the control and cocaine trials are classified as similar and placed in the same cluster by the dendrogram method in about 20% of the cases, regardless the number of clusters of the dendrogram (see Figure 6). This overlap suggests that there may be a common, invariant, part of the mathematical model that describes both control and cocaine trials. At the same time, the significant difference in the delay times between control and cocaine trials (see Figure 7) could lead to different phase space dynamics. This finding suggests that although there may be some similarities between control and cocaine trials, the differences in the delay times could be interpreted as two different time scales for the two experiments. Additionally, the Frechet distance plots shown in Figure 8 provide an intuitive and global understanding of potentially similar time scales between different trials as represented by blue colors.

This study complements a previous Fourier-based analysis done by our group (Dilgen et al., 2013). For the Fourier-based analysis, power spectral densities before and during the optogenetic stimulation were computed. It was found that stimulation at 40 Hz significantly increased oscillations and induced a clear peak in the gamma range (see Figure 3 in Dilgen et al., 2013). The effect of cocaine in the Fourier-based analysis was reflected in a decrease in bandwidth of induced oscillations (see Figure 4 in Dilgen et al., 2013). As a result, it was concluded that the main effect of cocaine is that “it increases the entrainment of the laser-induced oscillation to the driving frequency, resulting in a very narrow-bandwidth gamma oscillation centered at 40 Hz.”

Applications of nonlinear dynamics methods to neuroscience are based on the assumption that the central nervous system is a nonlinear dynamical system exhibiting deterministic chaos. The combination of the two words, “deterministic” and “chaotic,” seem unlikely, since we associate deterministic behavior of systems with predictable, well-behaved, responses governed by precise (deterministic) evolution equations whereas chaotic behavior is associated with unpredictable and “random” responses. More than a century ago, Poincaré shifted the paradigm by noticing that “…it may happen that small differences in the initial conditions produce very great ones in the final phenomena. A small error in the former will produce an enormous error in the latter. Prediction becomes impossible, and we have the fortuitous phenomenon” (Poincare, 1920). Poincaré showed that the three celestial bodies problem, although mathematically described by Newton's law of gravity, has “chaotic” behavior determined by initial conditions, i.e., sometimes small difference in initial conditions (positions and velocities of the three bodies) remain small and produce a well-behaved solution whereas for other infinitesimally close initial conditions the trajectories diverge exponentially. Such sensitivity of complex, nonlinear, systems to small variations in initial conditions are particularly important in computational neuroscience since the evolution equations are solved using digital computers that can only represent data with a finite precision. The nonlinearity and sensitivity to initial conditions is ubiquitous in neuroscience (Faure and Korn, 2001) and has been captured starting with early models, such as Hindmarsh and Rose model (Hindmarsh and Rose, 1984), which can produce a “chaotic” looking spike train that is actually deterministic. The interest in deterministic chaos and nonlinear dynamics is also clinically motivated as it has been shown that some nonlinear dynamics measures are very effective in detecting pathological conditions such as epileptic seizures, coma and dementia from electroencephalogram (EEG) recordings (Galka, 2000; Müller et al., 2001; Lehnertz, 2008). Others related the unfolding rate of the system attractor obtained with delay-embedding to different pathological cases of epilepsy (Pravitha et al., 2001), or used delay-embedding to filter various EEG artifacts (Anderson et al., 2006). Another important clinical application of delay-embedding include the detection of cardiac arrhythmia (Ravelli and Antolini, 1992; Richter and Schreiber, 1998) or the correlation between anxiety and electrocardiogram complexity (Radhakrishna and Vikram, 2001).

Other optogenetic modeling studies used a bottom-up approach in which relevant brain areas subject to optogenetic stimulation, such as the subthalamic nucleus (STN)—external Globus Pallidus (GPe) subnetwork, are modeled with conductance-based model neurons (Ratnadurai-Giridharan et al., 2017). Although limited to only ten STN and ten GPe neurons, the model seems to capture patterns of synchronized oscillatory activity observed in Parkinsonian patients. Their numerical simulations show that optogenetic inhibition is more effective than electrical deep brain stimulation (DBS) (Rosa et al., 2012; Wichmann and DeLong, 2016). While further studies are needed, a data-driven model obtained with the delay-embedding method could provide subject-tailored clinical support for alternative optogenetic solutions to DBS.

Other recent studies used a combination of delay embedding (Kantz and Schreiber, 1997; Hegger et al., 1999) and statistical learning theory (Hastie et al., 2001). The method was applied to a different data structure than ours, i.e., to analyze multiple single-unit recordings from the rat anterior cingulate cortex while the animals performed decision-making tasks in a radial-arm-maze (see Balaguer-Ballester et al., 2011 for details), or from the medial prefrontal cortex of rats while the animals performed a foraging task guided by working memory (Lapish et al., 2015). The authors augmented the embedding space with extra dimensions represented by interactions between units' firing rates to account for neuronal cross-correlations. In their study, the dimension of the embedding space (10^6^) was significantly larger than in our case. To handle the statistical analysis in such a high dimensional space, they used kernel-methods (Hastie et al., 2001). To visualize the phase space trajectories, they used a kernel-PCA (Principal Component Analysis) (Scholkopf et al., 1998; Zheng et al., 2005) by only retaining the three most variance-explaining dimensions of the neural dynamics. PCA algorithms find directions which have minimal reconstruction error by describing as much variance of the data as possible with a (relatively) small number of orthogonal directions. To further aid with visual interpretation of the data, they also projected neural dynamics onto a 3-dimensional space using a Fisher discriminant analysis (FDA) technique (Fisher, 1936; Mika et al., 1999; Sugiyama et al., 2009). FDA maximizes the separability of classes in a dataset by constructing projection vectors that maximize the scatter between the classes, while minimizing the scatter within each class (Duda et al., 2000). By augmenting the embedding space, the 3-dimensional visualizations seem to uniquely identify task-specific phases space attractors associated with different population states.

In this study, we corrected for the transient change in the LFPs by computing the amount of phase resetting required to maximize the correlation among trials. For truly nonstationary recordings, new methods have been suggested that range from machine learning to a novel class-trajectory coherence algorithm that can estimate the departures from deterministic nature in multi-attracting dynamics (Balaguer-Ballester et al., 2014). The method of class-trajectory coherence is particularly suitable for detecting subtile dynamical changes that are not detected by statistical moments and, therefore, when significant trends cannot be statistically proven (Balaguer-Ballester et al., 2014).

Our ultimate goal is to better understand the mechanisms of information processing and the role of the coherent 40 Hz oscillations. It is believed that this gamma rhythm of the brain is involved in higher cognitive function or consciousness (Mashour, 2006; Lee et al., 2009). The rhythm supports information integration across different areas of the brain and helps binding neural processes that generate consciousness as shown both experimentally (Tononi et al., 1998; Tononi and Sporns, 2003) and through computer modeling (Hauptmann et al., 2005). Loss of consciousness has been revealed by decoherence of gamma rhythm (John et al., 2001; Lee et al., 2009). The delay-embedding method has been previously used to investigate the change in the embedding dimension and lag times due to anesthetics (Walling et al., 2006; Lee et al., 2009). The “cognitive rebinding” associated with the emergence from unconsciousness was associated with the significant change from an ordered to a chaotic reconstructed attractor. Since the mice in our experiments were awake during both control and cocaine experiments, we did not find such a dramatic change in the attractors' structure. However, the underlying dynamics has a faster time scale for cocaine experiments, a detail that is only captured by analyzing the lag times.

## 8. Conclusions

The activity of the medial prefrontal cortex in six mice systemically injected with cocaine (20 mg/ kg ip) was optogenetically perturbed with brief laser pulses. The permanent phase resetting induced by a light stimulus was removed using the pair correlations between recorded local field potentials. The phase-corrected trials were embedded in a three dimensional phase space using a delay embedding method. The main results are as follows: (1) The reconstructed attractors for cocaine trials are three-dimensional. (2) The 20% classification overlap between control and cocaine trials suggests a possible common, invariant, mathematical description of network activity. (3) At the same time, the cocaine dynamics is about three times faster than the control, suggesting different time scales for a possible mathematical model. Since previous experiments using EEGs for delay-embedding focused only on comparing the wakefulness vs. unconsciousness attractors, we can only speculate that a gradual increase of the anesthetic could be useful in revealing not just the topological, i.e., the appearance and dimensionality of the attractor, but also the dynamical aspects, i.e., the lag time distribution, of phase space activity. Similarly, we would like to devote future experimental studies to investigate the effect of cocaine dose on the phase space reconstruction. We hypothesize that increasing the cocaine dose should shorten the lag times to the point where neural activity leads to a dramatic change even in the topology of the attractors. Such a hypothesis aligns with the general consensus of a communication breakdown between different parts of the cerebral cortex as consciousness fades (Massimini et al., 2005; Ferrarelli et al., 2010).

## Author contributions

SO contributed to the analysis and interpretation of data, and wrote the manuscript. JI and JH contributed to delay-embedding numerical simulations and reviewed the manuscript. TT and AL contributed to the design of the work, performed the experiments, and reviewed the manuscript.

### Conflict of interest statement

The authors declare that the research was conducted in the absence of any commercial or financial relationships that could be construed as a potential conflict of interest.

## References

[B1] Abarbanel H. (Ed.). (1996). Analysis of Observed Chaotic Data. New York, NY: Springer.

[B2] Allsop S. A. Vander Weele C. M. Wichmann R. Tye K. M. (2014). Optogenetic insights on the relationship between anxiety-related behaviors and social deficits. Front. Behav. Neurosci. 8:241. 10.3389/fnbeh.2014.00241 25076878 PMC4099964

[B3] Anderson C. W. Knight J. N. O'Connor T. Kirby M. J. Sokolov A. (2006). Geometric subspace methods and time-delay embedding for eeg artifact removal and classification. IEEE Trans. Neural Syst. Rehabil. Eng. 14, 142–146. 10.1109/TNSRE.2006.875527 16792280

[B4] Aponte Y. Atasoy D. Sternson S. M. (2011). Agrp neurons are sufficient to orchestrate feeding behavior rapidly and without training. Nat. Neurosci. 14, 351–355. 10.1038/nn.2739 21209617 PMC3049940

[B5] Atasoy D. Betley J. N. Su H. H. Sternson S. M. (2012). Deconstruction of a neural circuit for hunger. Nature 488, 172–177. 10.1038/nature11270 22801496 PMC3416931

[B6] Balaguer-Ballester E. Lapish C. Seamans J. Durstewitz D. (2014). Attracting dynamics of frontal cortex ensembles during memory-guided decision-making. PLoS Comput. Biol. 7:e1002057. 10.1371/journal.pcbi.1002057 21625577 PMC3098221

[B7] Balaguer-Ballester M. B. Tabas-Diaz A. Budka M. (2011). Can we identify non-stationary dynamics of trial-to-trial variability?. PLoS Comput. Biol. 9:e95648. 10.1371/journal.pone.0095648 24769735 PMC4000201

[B8] Bannister A. P. (2005). Inter- and intra-laminar connections of pyramidal cells in the neocortex. Neurosci. Res. 53, 95–103. 10.1016/j.neures.2005.06.019 16054257

[B9] Birkelund Y. Johansen J. A. Hanssen A. (2004). High-precision surrogate data based tests for gaussianity and linearity of discrete time random processes, in 2004 12th European Signal Processing Conference. Vienna.

[B10] Booker S. A. Gross A. Althof D. Shigemoto R. Bettler B. Frotscher M.. (2013). Differential gabab-receptor-mediated effects in perisomatic- and dendrite-targeting parvalbumin interneurons. J. Neurosci. 33, 7961–7974. 10.1523/JNEUROSCI.1186-12.2013 23637187 PMC3814621

[B11] Busskamp V. Duebel J. Balya D. Fradot M. Viney T. J. Siegert S.. (2010). Genetic reactivation of cone photoreceptors restores visual responses in retinitis pigmentosa. Science 329, 413–417. 10.1126/science.1190897 20576849

[B12] Buzsáki G. Draguhn A. (2004). Neuronal oscillations in cortical networks. Science 304, 1926–1929. 10.1126/science.1099745 15218136

[B13] Caillec J.-M. L. Montagner J. (2013). Fusion of hypothesis testing for nonlinearity detection in small time series. Signal Process. 93, 1295–1307. 10.1016/j.sigpro.2012.11.001

[B14] Cantero J. Atienza M. (2005). The role of neural synchronization in the emergence of cognition across the wake-sleep cycle. Rev. Neurosci. 16, 69–83. 10.1515/REVNEURO.2005.16.1.69 15810655

[B15] Cardin J. A. Carlen M. Meletis K. Knoblich U. Zhang F. Deisseroth K.. (2009). Driving fast-spiking cells induces gamma rhythm and controls sensory responses. Nature 459, 663–667. 10.1038/nature08002 19396156 PMC3655711

[B16] Casanova M. Trippe J. (2009). Radial cytoarchitecture and patterns of cortical connectivity in autism. Philos. Trans. Roy. Soc. 364, 1433–1436. 10.1098/rstb.2008.0331 19528027 PMC2677589

[B17] Casdagli M. Eubank S. Farmer J. D. Gibson J. (1991). State space reconstruction in the presence of noise. Phys. D 51, 52–98. 10.1016/0167-2789(91)90222-U

[B18] Chen Y. Knight Z. A. (2016). Making sense of the sensory regulation of hunger neurons. BioEssays 38, 316–324. 10.1002/bies.201500167 26898524 PMC4899083

[B19] Cheng C.-H. Chan P.-Y. S. Niddam D. M. Tsai S.-Y. Hsu S.-C. Liu C.-Y. (2016). Sensory gating, inhibition control and gamma oscillations in the human somatosensory cortex. Sci. Rep. 6, 20437–20447. 10.1038/srep20437 26843358 PMC4740805

[B20] Cogranne R. Retraint F. (2013). Application of hypothesis testing theory for optimal detection of lsb matching data hiding. Signal Process. 93, 1724–1737. 10.1016/j.sigpro.2013.01.014

[B21] Compte A. Reig R. Descalzo V. F. Harvey M. A. Puccini G. D. Sanchez-Vives M. V. (2008). Spontaneous high-frequency (10–80 hz) oscillations during up states in the cerebral cortex *in vitro*. J. Neurosci. 28, 13828–13844. 10.1523/JNEUROSCI.2684-08.2008 19091973 PMC6671899

[B22] Contractor A. Klyachko V. Portera-Cailliau C. (2017). Altered neuronal and circuit excitability in fragile x syndrome. Neuron 87, 699–715. 10.1016/j.neuron.2015.06.017 26291156 PMC4545495

[B23] Costa M. Goldberger A. L. Peng C.-K. (2005). Broken asymmetry of the human heartbeat: loss of time irreversibility in aging and disease. Phys. Rev. Lett. 95:198102. 10.1103/PhysRevLett.95.198102 16384029

[B24] Danziger A. (2013). Discrete Frechet Distance. Available online at: https://www.mathworks.com/matlabcentral/fileexchange/31922-discrete-frechet-distance?focused=3785717&tab=function&requestedDomain=www.mathworks.com

[B25] DeFelipe J. Farinas I. (1992). The pyramidal neuron of the cerebral cortex: morphological and chemical characteristics of the synaptic inputs. Prog. Neurobiol. 39, 563–607. 10.1016/0301-0082(92)90015-7 1410442

[B26] Diks C. van Houwelingen J. Takens F. DeGoede J. (1995). Reversibility as a criterion for discriminating time series. Phys. Lett. A 201, 221–228.

[B27] Dilgen J. Tompa T. Saggu S. Naselaris T. Lavin A. (2013). Optogenetically evoked gamma oscillations are disturbed by cocaine administration. Front. Cell. Neurosci. 7:213. 10.3389/fncel.2013.00213 24376397 PMC3841795

[B28] Do Monte F. Quirk G. Li B. Penzo M. (2016). Retrieving fear memories, as time goes by? Mole. Psychiatry 21, 1027–1036. 10.1038/mp.2016.78 27217148 PMC4956525

[B29] Duda R. O. Hart P. E. Stork D. G. (2000). Pattern Classification. New York, NY: John Wiley and Sons.

[B30] Eiter T. Mannila H. (1994). Computing Discrete Frechet Distance. Technical Report. Technical University of Vienna and University of Helsinki.

[B31] Eleftheriou C. Cesca F. Maragliano L. Benfenati F. Maya-Vetencourt J. (2017). Optogenetic modulation of intracellular signalling and transcription: Focus on neuronal plasticity. J. Exp. Neurosci. 11:1179069517703354. 10.1177/1179069517703354 28579827 PMC5415353

[B32] Ethridge L. E. White S. P. Mosconi M. W. Wang J. Pedapati E. V. Erickson C. A.. (2017). Neural synchronization deficits linked to cortical hyper-excitability and auditory hypersensitivity in fragile x syndrome. Mol. Autism 8:22. 10.1186/s13229-017-0140-1 28596820 PMC5463459

[B33] Faure P. Korn H. (2001). Is there chaos in the brain? i. concepts of nonlinear dynamics and methods of investigation. Comptes Rendus de l'Académie des Sciences - Series III - Sciences de la Vie 324, 773–793. 10.1016/S0764-4469(01)01377-4 11558325

[B34] Feldman M. (1984). Imorphology of the neocortical neuron, in The Cerebral Cortex, eds Peters A. Jones E. G. (New York, NY: Plenum Press). 123–200.

[B35] Ferrarelli F. Massimini M. Sarasso S. Casali A. Riedner B. A. Angelini G.. (2010). Breakdown in cortical effective connectivity during midazolam-induced loss of consciousness. Proc. Natl. Acad. Sci. U.S.A. 107, 2681–2686. 10.1073/pnas.0913008107 20133802 PMC2823915

[B36] Fisher R. A. (1936). The use of multiple measurements in taxonomic problems. Ann. Eugen. 7, 179–188. 10.1111/j.1469-1809.1936.tb02137.x

[B37] Fraser A. M. Swinney H. L. (1986). Independent coordinates for strange attractors from mutual information. Phys. Rev. A 33, 1134–1140. 10.1103/PhysRevA.33.1134 9896728

[B38] Frechet M. (1906). Sur quelques points du calcul fonctionnel. Rendiconti del Circolo Mathematico di Palermo 22, 1–74.

[B39] Fuchs E. C. Zivkovic A. R. Cunningham M. O. Middleton S. LeBeau F. E. Bannerman D.. (2007). Recruitment of parvalbumin-positive interneurons determines hippocampal function and associated behavior. Neuron 53, 591–604. 10.1016/j.neuron.2007.01.031 17296559

[B40] Fujiwara-Tsukamoto Y. Isomura Y. (2008). Neural mechanism underlying generation of synchronous oscillations in hippocampal network. Brain Nerve 60, 755–762. 10.11477/mf.1416100308 18646615

[B41] Galarreta M. Hestrin S. (2001). Spike transmission and synchrony detection in networks of gabaergic interneurons. Science 292, 2295–2299. 10.1126/science.1061395 11423653

[B42] Galka A. (2000). Topics in Nonlinear Time Series Analysis: With Implications for EEG Analysis. River Edge, NJ: Advanced series in nonlinear dynamics (World Scientific).

[B43] Garcia A. O. M. Muller M. F. Schindler K. Rummel C. (2013). Genuine cross-correlations: Which surrogate based measure reproduces analytical results best? Neural Netw. 46, 154–164. 10.1016/j.neunet.2013.05.009 23751366

[B44] Gelder R. N. V. (2015). Photochemical approaches to vision restoration. Vision Res. 111, 134–141. 10.1016/j.visres.2015.02.001 25680758 PMC4444397

[B45] Gibson J. R. Bartley A. F. Hays S. A. Huber K. M. (2008). Imbalance of neocortical excitation and inhibition and altered up states reflect network hyperexcitability in the mouse model of fragile x syndrome. J. Neurophysiol. 100, 2615–2626. 10.1152/jn.90752.2008 18784272 PMC2585391

[B46] Gradinaru V. Mogri M. Thompson K. R. Henderson J. M. Deisseroth K. (2009). Optical deconstruction of parkinsonian neural circuitry. Science 324, 354–359. 10.1126/science.1167093 19299587 PMC6744370

[B47] Grassberger P. (1987). Evidence for climatic attractors. Nature 362:524 10.1038/326524a0

[B48] Guidotti A. Auta J. Davis J. M. Dong E. Grayson D. R. Veldic M.. (2005). Gabaergic dysfunction in schizophrenia: new treatment strategies on the horizon. Psychopharmacology 180, 191–205. 10.1007/s00213-005-2212-8 15864560

[B49] Halasy K. Buhl E. Lorinczi Z. Tamas G. Somogyi P. (1996). Synaptic target selectivity and input of gabaergic basket and bistratified interneurons in the ca1 area of the rat hippocampus. Hippocampus 6, 306–329. 8841829 10.1002/(SICI)1098-1063(1996)6:3<306::AID-HIPO8>3.0.CO;2-K

[B50] Hastie T. Tibshirani R. Friedman J. (2001). The Elements of Statistical Learning. New York, NY: Springer New York Inc.

[B51] Hatsopoulos N. G. Donoghue J. P. (2009). The science of neural interface systems. Annu. Rev. Neurosci. 32, 249–266. 10.1146/annurev.neuro.051508.135241 19400719 PMC2921719

[B52] Haubensak W. Kunwar P. S. Cai H. Ciocchi S. Wall N. R. Ponnusamy R.. (2010). Genetic dissection of an amygdala microcircuit that gates conditioned fear. Nature 468, 270–276. 10.1038/nature09553 21068836 PMC3597095

[B53] Hauptmann C. Popovych O. Tass P. A. (2005). Effectively desynchronizing deep brain stimulation based on a coordinated delayed feedback stimulation via several sites: a computational study. Biol. Cybernet. 93, 463–470. 10.1007/s00422-005-0020-1 16240125

[B54] Heath R. A. (Ed.). (2000). Nonlinear Dynamics: Techniques and Applications in Psychology. Mahwah, NJ: Psychology Press.

[B55] Hegger R. Kantz H. Schreiber T. (1999). Practical implementation of nonlinear time series methods: the tisean package. Chaos 9, 413–435. 10.1063/1.166424 12779839

[B56] Henry T.-R. Markram M. Yun W. Anirudh G. Gilad S. Caizhi W. (2004). Interneurons of the neocortical inhibitory system. Nat. Rev. Neurosci. 5, 793–807. 10.1038/nrn1519 15378039

[B57] Hill T. Lewicki P. (Eds.) (2005). Statistics: Methods and Applications. Tulksa, OK: StatSoft, Inc.

[B58] Hille B. (Ed.). (2001). Ion Channels of Excitable Membranes. Sunderland, MA: Sinauer Associates, Inc.

[B59] Hindmarsh J. L. Rose R. M. (1984). A model of neuronal bursting using three coupled first order differential equations. Proc. Roy. Soc. Lond. B Biol. Sci. 1222, 87–102. 10.1098/rspb.1984.0024 6144106

[B60] Hodgkin A. L. Huxley A. F. (1952). A quantitative description of ion currents and its applications to conduction and excitation in nerve membranes. J. Physiol. (Lond.) 117, 500–544. 12991237 10.1113/jphysiol.1952.sp004764 PMC1392413

[B61] Holzfuss J. Mayer-Kress G. (1986). An approach to error-estimation in the application of dimension algorithms, in Dimensions and Entropies in Chaotic Systems, Vol. 32 of Springer Series in Synergetics. ed Mayer-Kress G. (Berlin; Heidelberg: Springer-Verlag), 114–122. 10.1007/978-3-642-71001-8_15

[B62] Hong L. E. Summerfelt A. Mitchell B. D. McMahon R. P. Wonodi I. Buchanan R. W.. (2008). Sensory gating endophenotype based on its neural oscillatory pattern and heritability estimate. Arch. Gen. Psychiatry 65, 1008–1016. 10.1001/archpsyc.65.9.1008 18762587 PMC2774756

[B63] Iurilli G. Ghezzi D. Olcese U. Lassi G. Nazzaro C. Tonini R.. (2012). Sound-driven synaptic inhibition in primary visual cortex. Neuron 73, 814–828. 10.1016/j.neuron.2011.12.026 22365553 PMC3315003

[B64] Jennings J. H. Rizzi G. Stamatakis A. M. Ung R. L. Stuber G. D. (2013). The inhibitory circuit architecture of the lateral hypothalamus orchestrates feeding. Science 341, 1517–1521. 10.1126/science.1241812 24072922 PMC4131546

[B65] John E. Prichep L. Kox W. Valdés-Sosa P. Bosch-Bayard J. Aubert E.. (2001). Invariant reversible qeeg effects of anesthetics. Conscious. Cogn. 10, 165–183. 10.1006/ccog.2001.0507 11414713

[B66] Jung K.-Y. Kim J.-M. Kim D. W. (2003). Nonlinear dynamic characteristics of electroencephalography in a high-dose pilocarpine-induced status epilepticus model. Epilepsy Res. 54, 179–188. 10.1016/S0920-1211(03)00079-2 12837569

[B67] Kahana M. J. Sekuler R. Caplan J. B. Kirschen M. Madsen J. R. (1999). Human theta oscillations exhibit task dependence during virtual maze navigation. Nature 399, 781–784. 10.1038/21645 10391243

[B68] Kaiser J. Lutzenberger W. (2003). Induced gamma-band activity and human brain function. Neuroscientist 9, 475–484. 10.1177/1073858403259137 14678580

[B69] Kajikawa Y. Schroeder C. (2011). How local is the local field potential? Neuron 72, 847–858. 10.1016/j.neuron.2011.09.029 22153379 PMC3240862

[B70] Kambe J. Kakimoto Y. Araki O. (2015). Phase reset affects auditory-visual simultaneity judgment. Cogn. Neurodyn. 9, 487–493. 10.1007/s11571-015-9342-4 26379799 PMC4567997

[B71] Kana R. K. Libero L. E. Moore M. S. (2011). Disrupted cortical connectivity theory as an explanatory model for autism spectrum disorders. Phys. Life Rev. 8, 410–437. 10.1016/j.plrev.2011.10.001 22018722

[B72] Kantz H. Schreiber T. (Eds.). (1997). Non-linear Time Series Analysis. Cambridge: Cambridge University Press.

[B73] Katzner S. Nauhaus I. Benucci A. Bonin V. Ringach D. L. Carandini M. (2009). Local origin of field potentials in visual cortex. Neuron 61, 35–41. 10.1016/j.neuron.2008.11.016 19146811 PMC2730490

[B74] Kennel M. B. Brown R. Abarbanel H. D. I. (1992). Determining embedding dimension for phase-space reconstruction using a geometrical construction. Phys. Rev. A 45, 3403–3411. 10.1103/PhysRevA.45.3403 9907388

[B75] Kim K. Kim J.-H. Song Y.-H. Lee S.-H. (2017). Functional dissection of inhibitory microcircuits in the visual cortex. Neurosci. Res. 116, 70–76. 10.1016/j.neures.2016.09.003 27633836

[B76] King G. P. Jones R. Broomhead D. (1987). Phase portraits from a time series: A singular system approach. Nucl. Phys. 2, 379–390. 10.1016/0920-5632(87)90029-6

[B77] Kokaia M. Andersson M. Ledri M. (2013). An optogenetic approach in epilepsy. Neuropharmacology 69, 89–95. 10.1016/j.neuropharm.2012.05.049 22698957

[B78] Konstantinou K. I. (2002). Deterministic non-linear source processes of volcanic tremor signals accompanying the 1996 vatnajakull eruption, central iceland. Geophys. J. Int. 148, 663–675. 10.1046/j.1365-246X.2002.01608.x

[B79] Kravitz A. V. Freeze B. S. Parker P. R. L. Kay K. Thwin M. T. Deisseroth K.. (2010). Regulation of parkinsonian motor behaviours by optogenetic control of basal ganglia circuitry. Nature 466, 622–626. 10.1038/nature09159 20613723 PMC3552484

[B80] Kugiumtzis D. (2002). Surrogate data test on time series, in Modelling and Forecasting Financial Data, Vol. 2 of Studies in Computational Finance. eds Soofi A. Cao L. (Boston, MA: Springer), 267–282.

[B81] Kugiumtzis D. Tsimpiris A. (2010). Measures of analysis of time series (mats): a matlab toolkit for computation of multiple measures on time series data bases. J. Stat. Softw. Art. 33, 1–30. 10.18637/jss.v033.i05

[B82] Lacasa L. Nsnez Á. M. Roldán É. Parrondo J. M. R. Luque B. (2012). Time series irreversibility: a visibility graph approach. Eur. Phys. J. B85:217 10.1140/epjb/e2012-20809-8

[B83] Lagali P. S. Balya D. Awatramani G. B. Munch T. A. Kim D. S. Busskamp V.. (2008). Light-activated channels targeted to on bipolar cells restore visual function in retinal degeneration. Nat. Neurosci. 11, 667–675. 10.1038/nn.2117 18432197

[B84] Lapish C. C. Balaguer-Ballester E. Seamans J. K. Phillips A. G. Durstewitz D. (2015). Amphetamine exerts dose-dependent changes in prefrontal cortex attractor dynamics during working memory. J. Neurosci. 35, 10172–10187. 10.1523/JNEUROSCI.2421-14.2015 26180194 PMC4502258

[B85] Lee U. Mashour G. A. Kim S. Noh G.-J. Choi B.-M. (2009). Propofol induction reduces the capacity for neural information integration: implications for the mechanism of consciousness and general anesthesia. Conscious. Cogn. 18, 56–64. 10.1016/j.concog.2008.10.005 19054696

[B86] Lehnertz K. (2008). Epilepsy and nonlinear dynamics. J. Biol. Phys. 34, 253–266. 10.1007/s10867-008-9090-3 19669475 PMC2585629

[B87] Levy F. (2007). Theories of autism. Aust. New Zealand J. Psychiatry 41, 859–868. 10.1080/00048670701634937 17924239

[B88] Lewis D. A. Hashimoto T. (2007). Deciphering the disease process of schizophrenia: the contribution of cortical gaba neurons. Int. Rev. Neurobiol. 78, 109–131. 10.1016/S0074-7742(06)78004-7 17349859

[B89] Lewis D. A. Hashimoto T. Volk D. W. (2005). Cortical inhibitory neurons and schizophrenia. Nat. Rev. Neurosci. 6, 312–324. 10.1038/nrn1648 15803162

[B90] Liddle E. B. Price D. Palaniyappan L. Brookes M. J. Robson S. E. Hall E. L.. (2016). Abnormal salience signaling in schizophrenia: the role of integrative beta oscillations. Hum. Brain Mapp. 37, 1361–1374. 10.1002/hbm.23107 26853904 PMC4790909

[B91] Lin D. Boyle M. P. Dollar P. Lee H. Lein E. S. Perona P.. (2011). Functional identification of an aggression locus in the mouse hypothalamus. Nature 470, 221–226. 10.1038/nature09736 21307935 PMC3075820

[B92] Liu X. Ramirez S. Pang P. T. Puryear C. B. Govindarajan A. Deisseroth K.. (2012). Optogenetic stimulation of a hippocampal engram activates fear memory recall. Nature 484, 381–385. 10.1038/nature11028 22441246 PMC3331914

[B93] Luczak A. Bartho P. Marguet S. L. Buzsaki G. Harris K. D. (2007). Sequential structure of neocortical spontaneous activity *in vivo*. Proc. Natl. Acad. Sci. U.S.A. 104, 347–352. 10.1073/pnas.0605643104 17185420 PMC1765463

[B94] Mashour G. A. (2006). Integrating the science of consciousness and anesthesia. Anesthes. Analges. 103, 975–982. 10.1213/01.ane.0000232442.69757.4a 17000815

[B95] Massimini M. Ferrarelli F. Huber R. Esser S. K. Singh H. Tononi G. (2005). Breakdown of cortical effective connectivity during sleep. Science 309, 2228–2232. 10.1126/science.1117256 16195466

[B96] Mehring C. Rickert J. Vaadia E. de Oliveira S. C. Aertsen A. Rotter S. (2003). Inference of hand movements from local field potentials in monkey motor cortex. Nat. Neurosci. 6, 1253–1254. 10.1038/nn1158 14634657

[B97] Melchitzky D. S. Lewis D. A. (2003). Pyramidal neuron local axon terminals in monkey prefrontal cortex: differential targeting of subclasses of gaba neurons. Cereb. Cortex 13, 452–460. 10.1093/cercor/13.5.452 12679292

[B98] Mercier M. Foxe J. Fiebelkorn I. Butler J. Schwartz T. Molholm S. (2013). Auditory-driven phase reset in visual cortex: human electrocorticography reveals mechanisms of early multisensory integration. NeuroImage 79, 19–29. 10.1016/j.neuroimage.2013.04.060 23624493 PMC3677511

[B99] Michael A. K. Zoe M. C. (2006). Gamma and beta neural activity evoked during a sensory gating paradigm: effects of auditory, somatosensory and cross-modal stimulation. Clin. Neuropsychol. 117, 2549–2563. 10.1016/j.clinph.2006.08.003 PMC1773003 17008125

[B100] Micheva K. D. Wolman D. Mensh B. D. Pax E. Buchanan J. Smith S. J.. (2016). A large fraction of neocortical myelin ensheathes axons of local inhibitory neurons. eLife 5:e15784. 10.7554/eLife.15784 27383052 PMC4972537

[B101] Mika S. Ratsch G. Weston J. Scholkopf B. Mullers K. R. (1999). Fisher discriminant analysis with kernels, in Neural Networks for Signal Processing IX: Proceedings of the 1999 IEEE Signal Processing Society Workshop (Cat. No. 98TH8468) (Madison, WI), 41–48. 10.1109/NNSP.1999.788121

[B102] Mitzdorf U. (1987). Properties of the evoked potential generators: current source-density analysis of visually evoked potentials in the cat cortex. Int. J. Neurosci. 33, 33–59. 3610492 10.3109/00207458708985928

[B103] Müller V. Lutzenberger W. Pulvermüller F. Mohr B. Birbaumer N. (2001). Investigation of brain dynamics in parkinson's disease by methods derived from nonlinear dynamics. Exp. Brain Res. 137, 103–110. 10.1007/s002210000638 11310163

[B104] Muraki K. Tanigaki K. (2015). Neuronal migration abnormalities and its possible implications for schizophrenia. Front. Neurosci. 9:74. 10.3389/fnins.2015.00074 25805966 PMC4354421

[B105] O'Connell P. Woodruff P. Wright I. Jones P. Murray R. (1997). Developmental insanity or dementia praecox: was the wrong concept adopted? Schizophr. Res. 23, 97–106. 9061806 10.1016/S0920-9964(96)00110-7

[B106] Oprisan S. (2013). All phase resetting curves are bimodal, but some are more bimodal than others. ISRN Comput. Biol. 2013, 1–11. 10.1155/2013/230571

[B107] Oprisan S. (2017). A consistent definition of phase resetting using hilbert transform. Int. Scholar. Res. Notices Comput. Biol. 2017:10. 10.1155/2017/5865101 28553658 PMC5434474

[B108] Oprisan S. Austin D. (2017). A generalized phase resetting method for phase-locked modes prediction. PLoS ONE 12:e0174304. 10.1371/journal.pone.0174304 28323894 PMC5360347

[B109] Oprisan S. Canavier C. (2002). The influence of limit cycle topology on the phase resetting curve. Neural Comput. 14, 1027–2002. 10.1162/089976602753633376 11972906

[B110] Oprisan S. Prinz A. Canavier C. (2004). Phase resetting and phase locking in hybrid circuits of one model and one biological neuron. Biophys. J. 87, 2283–2298. 15454430 10.1529/biophysj.104.046193 PMC1304653

[B111] Oprisan S. Thirumalai V. Canavier C. (2003). Dynamics from a time series: can we extract the phase resetting curve from a time series? Biophys. J. 84, 2919–2928. 10.1016/S0006-3495(03)70019-8 12719224 PMC1302855

[B112] Oprisan S. A. Lynn P. E. Tompa T. Lavin A. (2015). Low-dimensional attractor for neural activity from local field potentials in optogenetic mice. Front. Comput. Neurosci. 9:125. 10.3389/fncom.2015.00125 26483665 PMC4591433

[B113] Orekhova E. V. Stroganova T. A. Nygren G. Tsetlin M. M. Posikera I. N. Gillberg C.. (2007). Excess of high frequency electroencephalogram oscillations in boys with autism. Biol. Psychiatry 62, 1022–1029. 10.1016/j.biopsych.2006.12.029 17543897

[B114] Osborne A. Provencale A. (1989). Finite correlation dimension for stochastic systems with power-law spectra. Phys. D 35, 357–381.

[B115] Osorio I. Frei M. G. (2009). Seizure abatement with single dc pulses: ias phase resetting at play? Int. J. Neural Syst. 19, 149–156. 10.1142/S0129065709001926 19575505

[B116] Packard N. H. Crutchfield J. P. Farmer J. D. Shaw R. S. (1980). Geometry from a time series. Phys. Rev. Lett. 45, 712–716. 10.1103/PhysRevLett.45.712

[B117] Parastarfeizabadi M. Kouzani A. Z. (2017). Advances in closed-loop deep brain stimulation devices. J. NeuroEng. Rehabil. 14:79. 10.1186/s12984-017-0295-1 28800738 PMC5553781

[B118] Paz J. T. Davidson T. J. Frechette E. S. Delord B. Parada I. Peng K.. (2013). Closed-loop optogenetic control of thalamus as a tool for interrupting seizures after cortical injury. Nat. Neurosci. 16, 64–70. 10.1038/nn.3269 23143518 PMC3700812

[B119] Peng Z. Zhang N. Wei W. Huang C. S. Cetina Y. Otis T. S.. (2013). A reorganized gabaergic circuit in a model of epilepsy: Evidence from optogenetic labeling and stimulation of somatostatin interneurons. J. Neurosci. 33, 14392–14405. 10.1523/JNEUROSCI.2045-13.2013 24005292 PMC3761049

[B120] Pesaran B. Pezaris J. Sahani M. Mitra P. Andersen R. (2002). Temporal structure in neuronal activity during working memory in macaque parietal cortex. Nat. Neurosci. 5, 805–811. 10.1038/nn890 12134152

[B121] Peter J. U. Wolf S. (2010). Abnormal neural oscillations and synchrony in schizophrenia. Nat. Rev. Neurosci. 11, 100–113. 10.1038/nrn2774 20087360

[B122] Poincare H. (1920). Science et Methode. Paris: Bibliotheque de Philosophie Scientifique (Ernet Flammarion).

[B123] Pravitha R. Indic P. Nampoori V. P. N. Pratap R. (2001). Effect of time scales on the unfolding of neural attractors. Int. J. Neurosci. 111, 175–186. 10.3109/00207450108994229 11912673

[B124] Provenzale A. Smith L. Vio R. Murante G. (1992). Distinguishing between low-dimensional dynamics and randomness in measured time series. Phys. D Nonlin. Phenom. 58, 31–49. 10.1016/0167-2789(92)90100-2

[B125] Radhakrishna K. R. Vikram K. Y. (2001). Decreased chaos and increased nonlinearity of heart rate time series in patients with panic disorder. Auton. Neurosci. 88, 99–108. 10.1016/S1566-0702(01)00219-3 11474552

[B126] Ramirez S. Tonegawa S. Liu X. (2014). Identification and optogenetic manipulation of memory engrams in the hippocampus. Front. Behav. Neurosci. 7:226. 10.3389/fnbeh.2013.00226 24478647 PMC3894458

[B127] Ratnadurai-Giridharan S. Cheung C. Rubchinsky L. (2017). Effects of electrical and optogenetic deep brain stimulation on synchronized oscillatory activity in parkinsonian basal ganglia. IEEE Trans. Neural Syst. Rehabil. Eng. 25, 2188–2195. 10.1109/TNSRE.2017.2712418 28600255

[B128] Ravelli F. Antolini R. (1992). Complex dynamics underlying the human electrocardiogram. Biol. Cybernet. 67, 57–65. 10.1007/BF00201802 1606244

[B129] Rensing L. Ruoff P. (2002). Temperature effect on entrainment, phase shifting, and amplitude of circadian clocks and its molecular bases. Chronobiol. Int. 19, 807–864. 10.1081/CBI-120014569 12405549

[B130] Richter M. Schreiber T. (1998). Phase space embedding of electrocardiograms. Phys. Rev. E 58, 6392–6398. 10.1103/PhysRevE.58.6392

[B131] Rippon G. Brock J. Brown C. Boucher J. (2007). Disordered connectivity in the autistic brain: challenges for the new psychophysiology. Int. J. Psychophysiol. 63, 164–172. 10.1016/j.ijpsycho.2006.03.012 16820239

[B132] Rivnay J. Wang H. Fenno L. Deisseroth K. Malliaras G. (2017). Next-generation probes, particles, and proteins for neural interfacing. Sci. Adv. 3:e1601649. 10.1126/sciadv.1601649 28630894 PMC5466371

[B133] Rodriguez M. Caruncho H. Costa E. Pesold C. Liu W. Guidotti A. (2002). In patas monkey, glutamic acid decarboxylase-67 and reelin mrna coexpression varies in a manner dependent on layers and cortical areas. J. Comp. Neurol. 451, 279–288. 10.1002/cne.10341 12210139

[B134] Roldán É. (2014). Dissipation and Kullback–Leibler Divergence. Cham: Springer International Publishing.

[B135] Rosa M. Giannicola G. Marceglia S. Fumagalli M. Barbieri S. Priori A. (2012). Chapter three - neurophysiology of deep brain stimulation. Internat. Rev. Neurobio. 107, 23–55. 10.1016/B978-0-12-404706-8.00004-8 23206677

[B136] Rotschafer S. Razak K. (2014). Auditory processing in fragile x syndrome. Front. Cell. Neurosci. 8:19. 10.3389/fncel.2014.00019 24550778 PMC3912505

[B137] Roux F. Uhlhaas P. (2014). Working memory and neural oscillations: alpha-gamma versus theta-gamma codes for distinct wm information? Trends Cogn. Sci. 18, 16–25. 10.1016/j.tics.2013.10.010 24268290

[B138] Sanchez-Vives M. McCormick D. (2000). Cellular and network mechanisms of rhythmic recurrent activity in neocortex. Nat. Neurosci. 3, 1027–1034. 10.1038/79848 11017176

[B139] Saraçli S. Doğan N. Doğan İ. (2013). Comparison of hierarchical cluster analysis methods by cophenetic correlation. J. Inequalities Appl. 2013:203 10.1186/1029-242X-2013-203

[B140] Scherberger H. Jarvis M. R. Andersen R. A. (2005). Cortical local field potential encodes movement intentions in the posterior parietal cortex. Neuron 46, 347–354. 10.1016/j.neuron.2005.03.004 15848811

[B141] Schiff S. J. Chang T. (1992). Differentiation of linearly correlated noise from chaos in a biologic system using surrogate data. Biol. Cybernet. 67, 387–393. 10.1007/BF00200982 1391112

[B142] Schmidt M. Mirnics K. (2015). Neurodevelopment, gaba system dysfunction, and schizophrenia. Neuropsychopharmacology 40, 190–206. 10.1038/npp.2014.95 24759129 PMC4262918

[B143] Schnitzler A. Gross J. (2005). Normal and pathological oscillatory communication in the brain. Nat. Rev. Neurosci. 6, 285–296. 10.1038/nrn1650 15803160

[B144] Scholkopf B. Smola A. Muller K.-R. (1998). Nonlinear component analysis as a kernel eigenvalue problem. Neural Comput. 10, 1299–1319. 10.1162/089976698300017467

[B145] Schreiber T. Schmitz A. (2000). Surrogate time series. Phys. D 142, 346–382. 10.1016/S0167-2789(00)00043-9

[B146] Schuster H. G. Just W. (Eds.). (2005). Deterministic Chaos: An Introduction, 4th, Revised and Enlarged Edition. Weinheim: WILEY-VCH Verlag GmbH and Co. KGaA.

[B147] Sen A. K. Litak G. Syta A. (2007). Cutting process dynamics by nonlinear time series and wavelet analysis. Chaos Interdiscipl. J. Nonlin. Sci. 17:023133. 10.1063/1.2749329 17614687

[B148] Small M. (2005). Applied Nonlinear Time Series Analysis: Applications in Physics, Physiology and Finance. London, UK: World Scientific.

[B149] Small M. Judd K. Mees A. (2001). Testing time series for nonlinearity. Statist. Comput. 11, 257–268. 10.1023/A:1016604405201

[B150] Sohal V. S. Zhang F. Yizhar O. Deisseroth K. (2009). Parvalbumin neurons and gamma rhythms enhance cortical circuit performance. Nature 459, 698–702. 10.1038/nature07991 19396159 PMC3969859

[B151] Sohal V. S. Zhang F. Yizhar O. Deisseroth K. (2016). Insights into cortical oscillations arising from optogenetic studies. Biol. Psychiatry 71, 1039–1045. 10.1016/j.biopsych.2012.01.024 22381731 PMC3361599

[B152] Stam C. Nicolai J. Keunen R. (1998). Nonlinear dynamical analysis of periodic lateralized epileptiform discharges. Clin. Electroencephalogr. 292, 101–105. 10.1177/155005949802900209 9571298

[B153] Sugiyama M. Idé T. Nakajima S. Sese J. (2009). Semi-supervised local fisher discriminant analysis for dimensionality reduction. Mach. Learn. 78:35 10.1007/s10994-009-5125-7

[B154] Sultan K. Brown K. Shi S.-H. (2013). Production and organization of neocortical interneurons. Front. Cell. Neurosci. 7:221. 10.3389/fncel.2013.00221 24312011 PMC3836051

[B155] Takahata K. Kato M. (2008). Neural mechanism underlying autistic savant and acquired savant syndrome. Brain Nerve. 60, 861–869. 10.11477/mf.1416100319 18646626

[B156] Takens F. (1981). Detecting strange attractors in turbulence, in Dynamical Systems and Turbulence, Warwick 1980, Vol. 898 of Lecture Notes in Mathematics. eds Rand D. Young L.-S. (Berlin Heidelberg: Springer), 366–381. 10.1007/BFb0091924

[B157] Tass P. A. (2000). Stochastic phase resetting: a theory for deep brain stimulation. Prog. Theor. Phys. Suppl. 139, 301–313. 10.1143/PTPS.139.301

[B158] Tass P. A. (2003). A model of desynchronizing deep brain stimulation with a demand-controlled coordinated reset of neural subpopulations. Biol. Cybernet. 89, 81–88. 10.1007/s00422-003-0425-7 12905037

[B159] Theiler J. (1990). Estimating fractal dimension. J. Opt. Soc. Am. A 7, 1055–1073. 10.1364/JOSAA.7.001055

[B160] Theiler J. Eubank S. Longtin A. Galdrikian B. Farmer J. (1992). Testing for nonlinearity in time series: the method of surrogate data. Phys. D 58, 77–94.

[B161] Tononi G. Edelman G. M. Sporns O. (1998). Complexity and coherency: integrating information in the brain. Trends Cognit. Sci. 2, 474–484. 10.1016/S1364-6613(98)01259-5 21227298

[B162] Tononi G. Sporns O. (2003). Measuring information integration. BMC Neurosci. 4:31. 10.1186/1471-2202-4-31 14641936 PMC331407

[B163] Tye K. M. Mirzabekov J. J. Warden M. R. Ferenczi E. A. Tsai H.-C. Finkelstein J.. (2013). Dopamine neurons modulate neural encoding and expression of depression-related behaviour. Nature 493, 537–541. 10.1038/nature11740 23235822 PMC4160519

[B164] Tye K. M. Prakash R. Kim S.-Y. Fenno L. E. Grosenick L. Zarabi H.. (2011). Amygdala circuitry mediating reversible and bidirectional control of anxiety. Nature 471, 358–362. 10.1038/nature09820 21389985 PMC3154022

[B165] Veit J. Hakim R. Jadi M. P. Sejnowski T. J. Adesnik H. (2017). Cortical gamma band synchronization through somatostatin interneurons. Nat. Neurosci. 20, 951–959. 10.1038/nn.4562 28481348 PMC5511041

[B166] Vladimirov I. G. Petersen I. R. (2010). Minimum relative entropy state transitions in discrete time systems with statistically uncertain noise, in 49th IEEE Conference on Decision and Control (CDC) (Atlanta, GA).

[B167] Walling M. Peter T. Hicks B. Kenneth N. (2006). Nonlinear changes in brain dynamics during emergence from sevoflurane anesthesiapreliminary exploration using new software. Anesthesiology 105, 927–935. 17065886 10.1097/00000542-200611000-00013

[B168] Weiss G. (1975). Time-reversibility of linear stochastic processes. J. Appl. Probabil. 12, 831–836.

[B169] Wichmann T. DeLong M. R. (2016). Deep brain stimulation for movement disorders of basal ganglia origin: restoring function or functionality? Neurotherapeutics 13, 264–283. 10.1007/s13311-016-0426-6 26956115 PMC4824026

[B170] Wilson N. R. Runyan C. A. Wang F. L. Sur M. (2012). Division and subtraction by distinct cortical inhibitory networks *in vivo*. Nature 488, 343–348. 10.1038/nature11347 22878717 PMC3653570

[B171] Winkler I. Panknin D. Bartz D. Muller K.-R. Haufe S. (2016). Validity of time reversal for testing granger causality. IEEE Trans. Signal Proces. 64, 2746–2760. 10.1109/TSP.2016.2531628

[B172] Woelders T. Beersma D. Gordijn M. Hut R. Wams E. (2017). Daily light exposure patterns reveal phase and period of the human circadian clock. J. Biol. Rhyt. 32, 274–286. 10.1177/0748730417696787 28452285 PMC5476188

[B173] Wykes R. C. Kullmann D. M. Pavlov I. Magloire V. (2016). Optogenetic approaches to treat epilepsy. J. Neurosci. Methods 260, 215–220. 10.1016/j.jneumeth.2015.06.004 26072246

[B174] Yuan G.-C. Lozier M. S. Pratt L. J. Jones C. K. R. T. Helfrich K. R. (2004). Estimating the predictability of an oceanic time series using linear and nonlinear methods. J. Geophys. Res. 109:C08002 10.1029/2003JC002148

[B175] Zamparo M. Baldovin F. Caraglio M. Stella A. L. (2013). Scaling symmetry, renormalization, and time series modeling: the case of financial assets dynamics. Phys. Rev. E 88:062808. 10.1103/PhysRevE.88.062808 24483512

[B176] Zeng X. Eykholt R. Pielke R. A. (1991). Estimating the lyapunov-exponent spectrum from short time series of low precision. Phys. Rev. Lett. 66, 3229–3232. 10.1103/PhysRevLett.66.3229 10043734

[B177] Zheng W. Zou C. Zhao L. (2005). An improved algorithm for kernel principal component analysis. Neural Process. Lett. 22, 49–56. 10.1007/s11063-004-0036-x

[B178] Zumbach G. (2009). Time reversal invariance in finance. Quant. Finance 9, 505–515. 10.1080/14697680802616712

[B179] Zumbach G. (2012). Discrete Time Series, Processes, and Applications in Finance. Berlin Heidelberg: Springer.

